# Adipocytes disrupt the translational programme of acute lymphoblastic leukaemia to favour tumour survival and persistence

**DOI:** 10.1038/s41467-021-25540-4

**Published:** 2021-09-17

**Authors:** Q. Heydt, C. Xintaropoulou, A. Clear, M. Austin, I. Pislariu, F. Miraki-Moud, P. Cutillas, K. Korfi, M. Calaminici, W. Cawthorn, K. Suchacki, A. Nagano, J. G. Gribben, M. Smith, J. D. Cavenagh, H. Oakervee, A. Castleton, D. Taussig, B. Peck, A. Wilczynska, L. McNaughton, D. Bonnet, F. Mardakheh, B. Patel

**Affiliations:** 1 Centre for Haemato-Oncology, Barts Cancer Institute, John Vane Science Centre, Charterhouse Square, Queen Mary University of London, London, UK; 2 BHF Centre for Cardiovascular Science, The Queen’s Medical Research Institute, Edinburgh BioQuarter, University of Edinburgh, Edinburgh, Scotland UK; 3 Centre for Molecular Oncology, Barts Cancer Institute, John Vane Science Centre, Charterhouse Square, Queen Mary University of London, London, UK; 4 Department of Haemato-Oncology, St Bartholomew’s Hospital, West Smithfield, London, UK; 5 Christie NHS Foundation Trust, Manchester, UK; 6 Haemato-oncology Unit, The Royal Marsden Hospital, Sutton, UK; 7 Centre for Tumour Biology, Barts Cancer Institute, John Vane Science Centre, Charterhouse Square, Queen Mary University of London, London, UK; 8 CRUK Beatson Institute, Glasgow, UK; 9 Institute of Cancer Sciences, University of Glasgow, Glasgow, UK; 10 Haematopoietic Stem Cell Laboratory, The Francis Crick Institute, London, UK

**Keywords:** Acute lymphocytic leukaemia, Cancer microenvironment, Acute lymphocytic leukaemia

## Abstract

The specific niche adaptations that facilitate primary disease and Acute Lymphoblastic Leukaemia (ALL) survival after induction chemotherapy remain unclear. Here, we show that Bone Marrow (BM) adipocytes dynamically evolve during ALL pathogenesis and therapy, transitioning from cellular depletion in the primary leukaemia niche to a fully reconstituted state upon remission induction. Functionally, adipocyte niches elicit a fate switch in ALL cells towards slow-proliferation and cellular quiescence, highlighting the critical contribution of the adipocyte dynamic to disease establishment and chemotherapy resistance. Mechanistically, adipocyte niche interaction targets posttranscriptional networks and suppresses protein biosynthesis in ALL cells. Treatment with general control nonderepressible 2 inhibitor (GCN2ib) alleviates adipocyte-mediated translational repression and rescues ALL cell quiescence thereby significantly reducing the cytoprotective effect of adipocytes against chemotherapy and other extrinsic stressors. These data establish how adipocyte driven restrictions of the ALL proteome benefit ALL tumours, preventing their elimination, and suggest ways to manipulate adipocyte-mediated ALL resistance.

## Abstract (web summary)

How the bone marrow microenvironment evolves during induction chemotherapy to facilitate acute lymphoblastic leukaemia (ALL) survival remains unclear. Here the authors show that adipocytes emergent during ALL therapy aid the survival of ALL cells through a restrictive effect on the ALL proteome.

## Introduction

Despite remarkable improvements in the treatment of paediatric ALL with cure attainable for the majority (>80%) of patients^1^, more than half of adults with ALL will relapse and die within 5 years of diagnosis despite achieving an excellent initial response to treatment^2^. These results indicate that whilst current treatments are effective at eradicating the bulk of the adult ALL tumour, complete disease eradication in these patients is rare and individuals remain at risk of disease recurrence. Thus, a central quest in adult ALL is to define the critical drivers of leukaemia cell persistence as a key strategy for overcoming eventual therapy failure. Clinically, the therapy-surviving leukaemia is characterized as the minimal residual disease (MRD), which strongly and independently predicts a high subsequent risk of disease recurrence^3–5^, emphasizing the key importance of these targets to ALL evolution. Hallmarks of therapy-latent MRD subclones include intrinsic chemoresistance, dormancy and long-term persistence as well as functional plasticity, as assessed experimentally^6,7^ and predicted clinically. However, the regulatory mechanisms underpinning these aberrant states are not completely understood. Of relevance here is the increasing understanding of the broad biological context of MRD subclone escape beyond that predicted by genotype and epigenetics alone^8,9^, raising interest in nongenetic drivers of this process^10^. The relevant player here is the bone marrow microenvironment (BMM), the primary residence of ALL; comprising a highly diverse network of cellular (mesenchymal stroma, osteoblasts and endothelial cells), soluble and structural factors that work together to coordinate and maintain haematopoietic stem cell (HSC) function^11^. To perform its role, the BMM must adapt to changing physiological contexts while still regulating and maintaining HSCs, emphasizing the principle that BMMs are inherently dynamic^12^^.^ This distinct property of the BM niche is co-opted by leukaemia cells to support developing tumours and to shaping the ecological competition between leukaemia and healthy haematopoiesis, a process driven by aberrant crosstalk between leukaemia cells and BM stroma^13–17^. Upon exposure to chemotherapeutic stress, BMMs undergo further adaptation^12,18,19^, highlighting the continuously evolving habitat in which leukaemia cells reside. These findings indicate that niches in the BMM may differ dynamically during primary disease and after chemotherapy treatment^18,19^. However, many studies fail to account for these niche dynamics explicitly and from the outset. Moreover, the appropriate context for MRD (<5% leukaemia blasts) is defined after the transition to morphological remission, yet the niche in this setting has yet to be fully defined. Where this paradigm has been applied, emergent and novel mechanisms of niche-resistance have been identified^14,20^, highlighting the fundamental importance of temporal dynamics in determining which events precipitate treatment failure.

We therefore hypothesized that the evolving course of BM niches from ALL disease through to remission-inducing chemotherapy would provide a tool for mapping dynamic parameters that temporally and biologically contribute to subclone-specific resistance. Here we show that the transition from disease to treatment and remission rebuilds the adipocytic BM niche, a major BMM implicated in ALL resistance, and demonstrate a previously unknown capacity of adipocytes to non-cell autonomously repress the ALL proteome as a mechanism for increasing ALL cell quiescence and multistress resistance that may ultimately contribute to leukaemia re-evolution.

## Results

### The adipocyte BM niche is dynamically remodelled during ALL pathogenesis and treatment

**Fig. 1:**
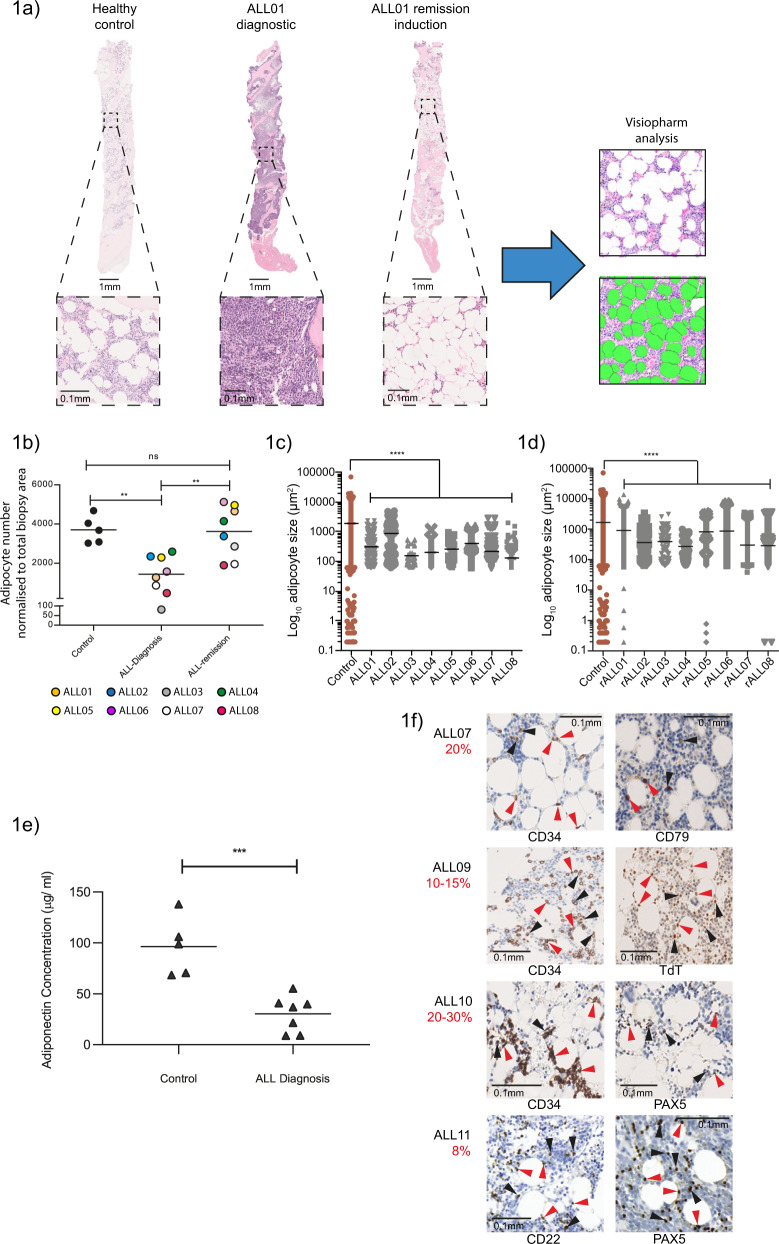
The adipocyte BM niche is dynamically remodelled during ALL pathogenesis and treatment. **a** H&E-stained human BM biopsies from healthy control, patient with ALL at diagnosis (ALL01 Diagnostic) and post remission induction treatment (ALL01 Remission Induction). Representative of five independent healthy BM biopsies and eight independent matched pairs of ALL diagnosis and remission biopsies analysed once/biopsy. Zoomed-in images of the boxed regions are presented below. Black arrowheads indicate BM adipocytes. Custom image analysis using Visiopharm software identifies individual BM adipocytes as green objects. **b** Adipocyte numbers quantified by Visiopharm analysis in healthy controls (*n* = 5) and paired ALL diagnosis vs ALL remission BM biopsies (*n* = 8). Each datapoint denotes an independent biopsy. Data are normalized to the size of the biopsy. **p < 0.01 by one-way ANOVA with Tukey’s multiple comparison test. **c** Adipocyte size quantified by Visiopharm analysis in healthy control (*n* = 5) and ALL diagnosis (*n* = 8, ALL01-ALL08) BM biopsies. Data points denote values for individual adipocytes (>45). The mean ± SEM is shown. Statistical significance was assessed by a Kruskal–Wallis test with Dunn’s multiple comparisons test (****p < 0.0001). **d** Adipocyte size quantified by Visiopharm analysis in healthy control (*n* = 5) and ALL remission (rALL) BM biopsies (*n* = 8). Data points denote values for individual adipocytes (>72). The mean ± SEM is shown. Statistical significance was assessed by a Kruskal–Wallis test with Dunn’s multiple comparisons test (****p < 0.0001). **e** Adiponectin concentrations in serum samples from healthy control vs ALL diagnosis BM (including ALL04, ALL17 and ALL21). Adiponectin was quantified using a commercial ELISA kit. Each datapoint denotes an independent BM serum sample. ***p < 0.0006 by a 2-sided unpaired *t* test. **f** Morphological evaluation of residual ALL disease in H&E-stained BM biopsies from four consecutively assessed patients with an incomplete response (<5% blasts) to remission induction chemotherapy. Individual images show ALL tumour-specific immunostaining. Red arrows indicate ALL blasts in close proximity to BM adipocytes while black arrows denote interstitially distributed ALL disease as assigned by 2 independent reviews. The percentage of residual ALL disease was obtained from clinical reports and is indicated in red text.

To probe the transitional nature of BMM, we compared the composition of matched BM biopsies taken at initial ALL diagnosis and after remission induction by gross histology in eight adult ALL patients. Our studies were limited to B lineage ALL (B-ALL), which accounts for >80% of the disease incidence^21^. Consistent with the assertion of niche evolution, we observed profound remodelling of the BMM accompanying ALL development and again after therapeutic intervention, most strikingly affecting adipocyte compartments, which were profoundly depleted in ALL-BMs compared to healthy controls, with apparent adipocyte reconstitution upon remission attainment (Fig. 1a). Adipocytes are microenvironmental cell types that have a well established role in ALL resistance^14,17,19,22–27^; however, the niche dynamics leading to this stage have been minimally explored, prompting us to investigate these BM components further. We first applied custom image analysis to rigorously define and quantify the temporal changes in BM adipocyte stucture. These assessments revealed a profound loss of adipocyte numbers (Fig. 1b) as well as  major reductions in adipocyte size (Fig. 1c) at ALL diagnosis,  implicating both of  these factors in the profound adipocyte suppression observed under this condition. In contrast, remission biopsies demonstrated an evolution to normal adipocyte numbers (Fig. 1b), indicating a return of adipocyte population homeostasis upon successful therapy. These outcomes differ from those reported in AML, where chemotherapy leads to an inhibition of adipogenesis in the BM^28^. Notably, remission states did not fully normalize adipocyte size (Fig.1d and Table S1a), suggesting that BM-adipocyte numbers in particular display striking sensitivity to the presence of ALL. Importantly, ALL-associated adipocyte suppression was unrelated to BM cellularity, confirming that changes in adipocyte population activity reflected absolute versus proportional losses. (Supplementary Fig. S1a and S1b). We additionally measured adiponectin production in the marrow plasma from ALL patients as a functional marker of BM adiposity^29^, and showed it was reproducibly decreased (Fig. 1e), consistent with the population-level losses observed histologically. Altogether, these data clarify that the BM-adipocyte stroma is not a prominent component of the primary tumour microenvironment but the emergent niche associated with the remission response.

To investigate how these niche dynamics relate to clinical chemoresistance, we examined ALL cell interactions in BM trephines from patients who failed to achieve complete remission (>5% blasts morphologically after induction therapy). These analyses revealed two consistent patterns across independent BM biopsies; chemoresistant ALL cells were distributed either interstitially throughout the marrow (Fig. 1f) or in close neighbouring proximity to adipocytes. Thus, while chemoresistance is spatially heterogeneous within the BM niche, as previously suggested^17^, distinct physical interactions with the surrounding adipocyte stroma are recurring features of drug survival in ALL.

### ALL corrupts the functioning and lineage priming of the adipocyte precursor mesenchymal stromal-cell (MSC) population

**Fig. 2:**
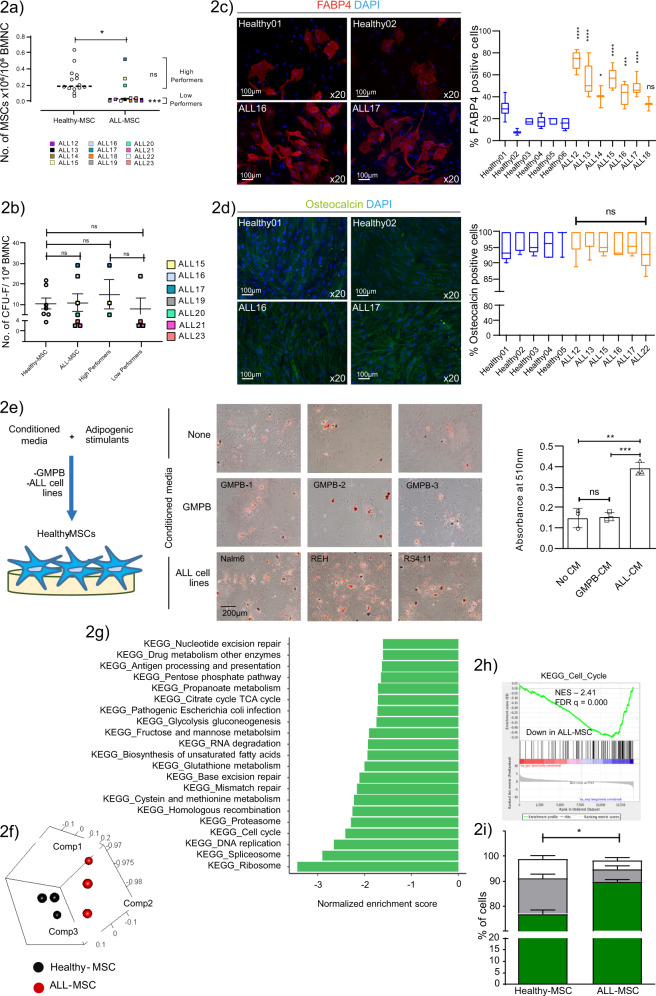
ALL corrupts the functioning and lineage priming of the adipocyte precursor mesenchymal stromal-cell (MSC) population. **a** Growth outcomes of ALL-MSCs (ALL12-ALL23) at P1. Each datapoint denotes an independent BM. ALL-MSCs were divided into high and low performers defined by the group mean. Horizontal line denotes the median *p < 0.0132, ***p < 0.001. **b** CFU-F numbers from seven independent ALL (ALL15-17, ALL19-21 and ALL23) and healthy-BMs after +10 days under MSC differentiation conditions. **c** Adipocyte-specific FABP4 staining in ALL vs healthy-MSCs (left) and FABP4 fluorescence quantification (right). Images are representative of seven and six independent BM samples, respectively analysing 100 cells/sample. **d** Osteocalcin staining in ALL vs healthy-MSCs (left) and osteocalcin fluorescence quantification (right). Images are representative of six and five independent BM samples, respectively. For (**c**) and (**d**) comparisons are between each individual ALL-MSC and the aggregated mean of healthy-MSCs. Horizontal line denotes the median; boxes extend from the 25th to the 75th percentile. **e** Representative micrographs (left) showing intracellular lipid staining with oil red (magnification, ×10; scale bar, 200 µm) following in vitro adipogenic differentiation in the absence or presence of conditioned media (CM) from cultured GMPB, from *n* = 3 biologically independent samples or from ALL cell lines (Nalm-6, REH and RS4;11). Oil red staining was quantified in three independently assayed wells/condition from one experiment. **p = 0.002, ***p = 0.0004. **f** Principal component analysis of global RNA-seq data from ALL-MSCs (ALL12-14) and age-matched healthy-MSCs (*n* = 3). Each dot represents an individual BM-MSC. **g** GSEA comparing RNA-seq**-**generated global transcriptomes of ALL-MSCs (*n* = 3) vs healthy-MSCs (*n* = 3) by KEGG pathway annotation. Significance was assigned by FDR *q*-value<0.05. Bars in green correspond to downregulated pathways. **h** Gene set enrichment plot for the KEGG cell cycle pathway demonstrating significant downregulation of these gene sets in ALL-MSCs. **i** Ki67 cell cycle analysis of ALL-MSCs (*n* = 4) vs healthy-MSCs (*n* = 4). Statistical comparisons are between each cell cycle stage. *p = 0.029. Unless otherwise stated, all data are presented as mean values ± SEM values of independent experiments (*n* = 4 in (**a**), *n* = 3 in (**b**), *n* = 1 in (**e**) and *n* = 3 in (**i**). Statistical significance was assessed using Kruskal–Wallis test with Dunn’s multiple comparisons test (**a**–**d**),  2-sided unpaired *t* tests (**e**), two-sided Mann–Whitney *U* test (**i**). *p < 0.05, **p < 0.01,***p < 0.001, ****p < 0.0001) or the precise *p*-value where indicated. ns, not significant. NES, normalized enrichment score; FDR false discovery rate.

To investigate the mechanism by which adipocytes are depleted in ALL-BMMs, we performed a global evaluation of the BM-MSC axis, hypothesizing that these precursors for BM-adipocyte generation were disrupted by ALL disease. We found that MSCs could be generated with similar success from ALL vs healthy control BM (Table Supplementary 2a) and that the BM-MSC phenotype was highly conserved in ALL disease (Supplementary Fig. 2a)^30^ confirming these isolates were bona fide MSCs. We further assessed the growth properties of ALL-MSCs using the growth rate in vitro as a measure of proliferative potential and where cell numbers permitted functional assessment of clonogenic capacity. We observed a nonuniform growth response in ALL-MSCs, reflecting expected differences in tumour and host biology. We therefore dichotomized growth data into high and low performers defined by the mean number of ALL-MSCs (Fig. 2a). High performers constituted a small subgroup (3/12, 25%) whose growth activity and CFU-F were indistinguishable from healthy BM-MSCs; however, most ALL-MSCs (9/12, 75%) demonstrated significantly reduced growth capacity (mean ± SEM: ALL-MSCs 0.095 ± 0.046 vs healthy-MSCs 0.254 ± 0.041), although interestingly, had  retained their CFU-F forming potential (Fig. 2b), suggesting that the low expansion capacities of these ALL-MSCs were principally driven by defective colony growth vs colony formation. We next assessed the multidifferentiation potential of ALL-MSCs following in vitro developmental induction. Unexpectedly, we found that ALL-MSCs had a significantly enhanced capacity for adipogenic differentiation (2.87 ± 0.29-fold increase; *n* = 7; ***p = 0.0003), as determined by cell staining for adipocyte-specific fatty acid binding 4 (FABP4) (Fig. 2c), without any reciprocal disturbance in osteogenesis (Fig. 2d), indicating that ALL pathogenesis significantly affects the lineage priming of MSCs in an adipocyte-biased manner. As the adipogenic potential of BM-MSCs in AML disease and similar conditions has been shown to decrease^31,32^, although not consistently^33^, this raises the possibility that altered differentiation fates in ALL-MSCs may represent a tumour-specific interplay.

To test the possibility that ALL cells may directly induce changes in their surrounding stromal cells, we tested the effect of leukaemic serum on healthy age-matched MSC stroma (Fig. 2e). We found that enhanced adipocyte commitment could be reproduced by exposing healthy MSCs to leukaemic but not HSC-enriched GCSF-mobilized peripheral blood (GMPB)-conditioned media, confirming a direct role of leukaemia specific factors in altering the cell fate determination of  BM mesenchymal stroma.

Next, to gain further insight into the transcriptional programmes underlying the altered functionality and commitment of ALL-MSCs, we performed RNAseq studies on MSCs from three healthy and three low-performer ALL-BMs (ALL12, ALL13, ALL22) at the P4 expansion stage. Three-dimensional principal component visualization clearly differentiated ALL-MSCs from their healthy counterparts based on log_2_ transformed unsupervised global profiles suggestive of disease-specific transcriptional modulation (Fig. 2f). Further gene set enrichment analysis (GSEA) of these global RNA-seq datasets identified genes involved in cellular metabolism and multiple aspects of cell cycle function (Fig. 2g, h) as preferentially downregulated in ALL-MSCs, whereas no gene sets were identified as significantly (FDR *q*-value<0.05) upregulated. Notably, the BURTON_ADIPOGENESIS KEGG pathway, which specifies a committed gene program for adipocyte differentiation, was not significantly enriched in the ALL-MSCs, confirming i) that ALL-MSC isolates were not adipocyte precursor cells and ii) that ALL-MSCs are poised rather than committed to programmed adipocyte differentiation. To validate the findings of GSEA suggesting attenuated cell cycle function in ALL-MSCs, we performed a functional analysis in four low-performer ALL-MSCs (ALL12-14, ALL22) at the P4 expansion stage. This analysis confirmed that ALL-MSCs display slower proliferation kinetics (Fig. 2i), providing a potential mechanistic basis for their impaired growth function in vitro.

Collectively, our data suggest that ALL disease corrupts regenerative growth and the differentiation of MSCs. Such perturbations may lead to a reduced number of MSC precursors inhabiting the BM of ALL, which may account for disease-associated loss of adipocyte stroma. However, accumulation of adipocyte-primed MSCs, in the context of adipocyte-depleted ALL-BM, leads to the proposal that their differentiation must be stalled, most likely through a blast-specific mechanism, an effect that likely is reversed upon ALL clearance.

### Adipocytes create a tumour-suppressive niche in ALL

**Fig. 3:**
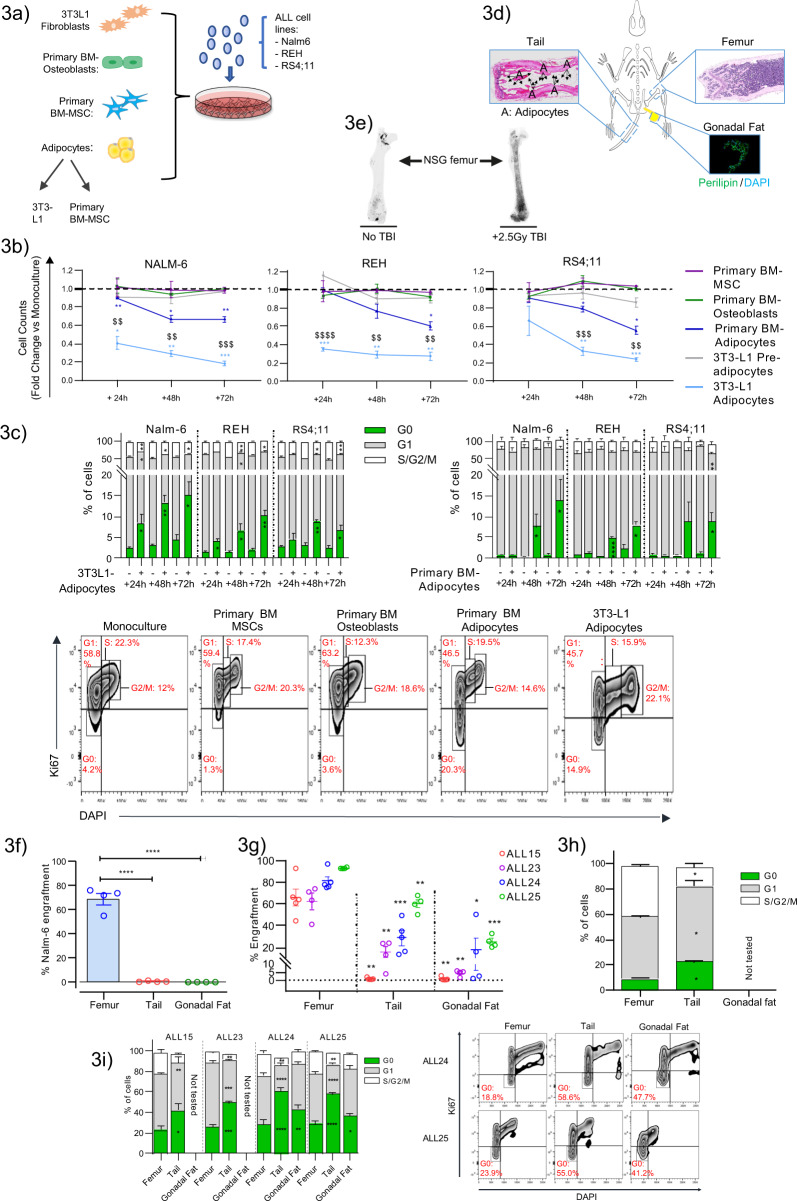
Adipocytes create a tumour-suppressive niche in B-ALL. **a** Schematic of the experimental setup to assess functional interactions between ALL and adipocyte niches. **b** In vitro growth of ALL cell lines (Nalm-6, REH and RS4;11) in adipocyte and unrelated stromal environments over time. Primary BM-MSCs from three independent healthy donors were evaluated alongside their corresponding osteoblast and adipocyte derivative. **c** Frequency of quiescent (G0) and cycling (G1 and S/G2/M) populations in ALL cells after monoculture (−) vs adipocyte coculture (+) from experiments described in (**b**). Bottom panel shows representative Ki67/DAPI staining in CD19 + -gated Nalm-6 cells at +72 h. Percentage of cells in each phase of the cell cycle is shown in red. **d** Schematic showing the different microenvironments assayed for human ALL xenotransplantation studies. BM from the tail and gonadal fat are designated adipocyte-rich niches, whereas femoral BM is adipocyte poor. Arrows point to individual adipocytes. **e** Osmium-stained and micro-CT-imaged BM adipocytes in whole femurs from NSG mice at +10 days. BM-adipocyte production was stimulated by sublethal (2.5 Gy) total body irradiation and served as a positive control. **f**, **g** Engraftment outcomes of Nalm-6 (CD19+/CD10+) and four independent primary B-ALL (CD19+CD45+) tumours following tail vein IV injection in distinct in vivo niches. Statistical comparisons are with femur. **h**, **i** Comparison of cell cycle characteristics in Nalm-6 and primary B-ALL xenografts respectively from experiments described in (**f**, **g**). Not tested indicates failure to perform robust cell cycle analysis due to low cell recovery. The panel on the right shows representative Ki67/DAPI staining in CD19+ gated primary B-ALL xenografts in the indicated niches. The percentage of cells in the G0 phase is shown in red. Data are presented as mean values ± SEM values of independent experiments (*n* = 3 with 2–3 replicates in (**b**), *n* = 1 in (**f**, **h**) with *n* = 3 mice and *n* = 3 in (**g**, **i**) *n* = 4–5 mice. Statistical significance was assessed using 2-sided unpaired *t* tests (**b**, **c**, **i:** ALL15 and 23). ANOVA with Dunnett’s multiple comparisons test (**f**, **g**, **i:** ALL24 and ALL25), two-sided Mann–Whitney *U*-tests (**h**) (*p < 0.05, **p < 0.01, ***p < 0.001, ***p < 0.001). ^$$^*p* < 0.01, ^$$$^*p* < 0.001 for primary vs 3T3-L1 adipocytes. Only statistically significant comparisons are indicated (**b**, **c**).

To explore the possibility that variations of adipocyte activity within the BMM contribute a meaningful pathophysiological role vs merely serving as a by-product of the leukaemic process, functional studies were performed to establish the effects of adipocyte stroma on ALL cells using an in vitro coculture strategy employing primary human adipocytes differentiated from healthy BM-MSCs or adipocytes derived from murine 3T3-L1 cell lines^34^ together with ALL cell lines; Nalm-6, REH and RS4;11 (Fig. 3a).

Surprisingly, we observed that both 3T3-L1 and primary BM-MSC adipocytes robustly impaired the growth capacity of independent ALL cell lines with high reproducibility compared to log phase monocultures (Fig. 3b), with evidence of a dosage-dependent effect, as indicated by lower relative growth rates in 3T3-L1 adipocyte conditions that have more complete adipocyte conversion (Supplementary Fig. 3a). Notably, basal growth of ALL cell lines was unaffected by coculture with primary osteoblasts, primary BM-MSCs and 3T3-L1 preadipocytes, confirming that adipocytes inhibited in vitro ALL growth in a tissue-specific manner and that this was a direct causal effect. Mechanistically, we confirmed that adipocyte niches did not lead to appreciable apoptosis (Supplementary Fig. 3b) but instead antagonized cell cycle progression, resulting in reproducible expansion of a quiescent cell pool accompanied by variable reductions in S/G2/M progression (Fig. 3c). Thus, interaction with adipocyte niches in vitro suppresses the constitutive proliferation of ALL cells and lowers cell cycle transit in a causal manner. Furthermore, these data confirm that in vitro murine 3T3-L1-derived adipocytes^34^ robustly recapitulate key responses of primary BM-adipocyte tissue cultures thereby establishing the reliability of these models as an experimental system. As further validation, adipocytes derived from BM-MSCs obtained after remission induction were also assessed and were shown to induce similar outcomes to those of healthy BM-MSC primary adipocytes (Supplementary Fig. 3c), thus extending the relevance of the described effects to the post-treatment niche setting.

To investigate the importance of these data in an in vivo context, experiments were conducted ﻿comparing in vivo ALL cellular response in adipocyte-rich (tail-BM and gonadal fat) vs adipocyte-poor (femur) tissues using xenotransplantation assays of the Nalm-6 ALL cell line and four independant  primary B-ALL tumours (Fig. 3d). We included gonadal fat based on the finding that 3T3-L1 adipocytes, a non-BM restricted tissue, showed no difference in adipocyte functionality in terms of executed effects on ALL cells. We first confirmed that NSG mice  have low BM-adipocyte abundance in femoral BM, as demonstrated by osmium tetroxide staining (Fig. 3e), validating the study of femoral BM as a model of adipocyte-poor marrow. We found that both the xenotransplanted Nalm-6 ALL cell line (Fig. 3f) and primary ALL cells from four genetically distinct tumours (Fig. 3g) had significantly less leukaemia cell engraftment in all adipocyte-rich tissues at 3-4 and 6-8 weeks following transplantation respectively when compared to adipocyte-poor femoral BM, which demonstrated robust leukaemia engraftment at this time. Thus, consistent with the functional outcomes in vitro (Fig. 3b), ALL cells in vivo demonstrate limited expansion in adipocyte niches. Further direct phenotypic characterization of engrafted tumours confirmed that ALL cells resident in adipocyte niches were marked by increased cellular quiescence (Fig. 3h, i) and reduced cell cycle G1/S/M status, substantiating the concept that adipocytes support tumour dormancy states. These results represent a broader biological relationship with ALL than previously described^24^ and are in line with reported effects in HSCs^35^.

Taken together, these data conceptualize adipocytes as key drivers of ALL cell plasticity and switchers of cellular fate from constitutive proliferator status to a phenotype characterized by proliferative quiescence. These data support the concept that changes in BM-adipocyte population dynamics facilitate ALL in distinct ways. Compromised adipocyte activity, possibly through ALL cells actively deregulating adipocyte generation, eliminates a key inhibitory niche for ALL, thereby enabling the emergence of leukaemia. However, reinstated adipocyte activity during the treatment response evolves a pro-tumour dormancy setting that could participate in antagonizing chemotherapy targeting.

### Adipocyte niches restrict protein synthesis in ALL via non-canonical factors

We next sought to define the mechanism(s) underlying the dormancy/growth suppressive response of ALL cells to microenvironmental adipocytes. We selected 3T3-L1 adipocyte models to initialize these studies based on their strong recapitulation of the BM-adipocyte interplay (Fig. 3b, c) and the ability to control for major confounding effects, e.g., nonadipocyte stromal-cell contamination (Supplementary Fig. 3a), thus, overcoming the principal limitation of in vivo adipocyte niche systems^36^.

**Fig. 4:**
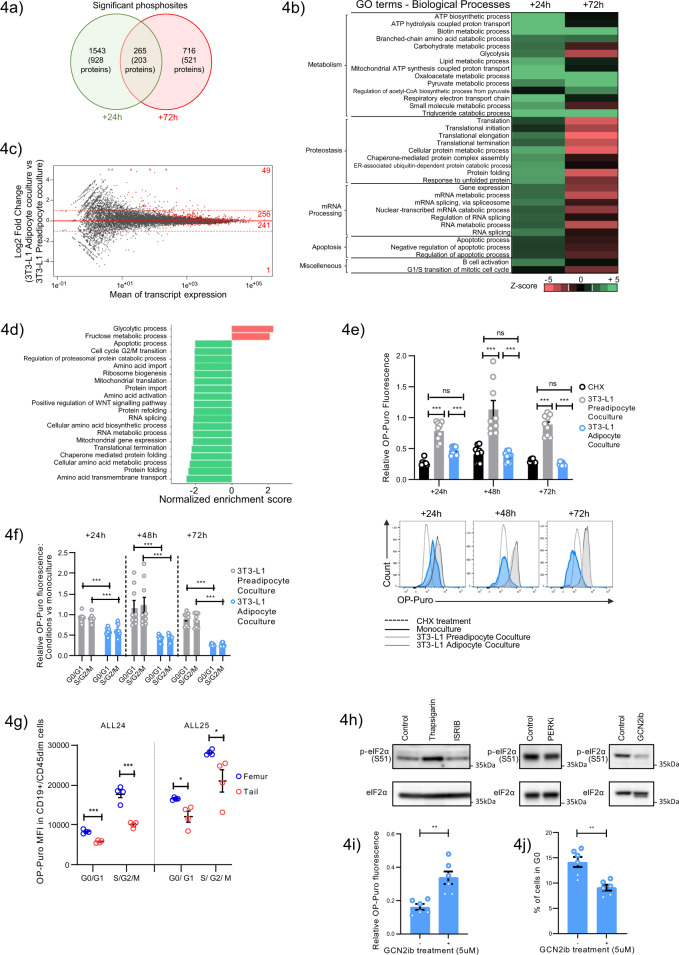
Adipocyte niches restrict protein synthesis in ALL via non-canonical factors. **a** Venn diagram of significant phosphosites identified in Nalm-6 cells cultured with 3T3-L1 adipocytes at +24 (green) and +72 h (red). Bracketed values denote the corresponding number of proteins. **b** Pathway analysis of the altered phosphoproteins from (**a**). Z-score, red: ≤−1.5, pathway underrepresented; green: ≥+1.5, pathway overrepresented. GO terms are grouped into categories that were hand curated. **c** Differential transcription level (log_2_) between Nalm-6 cells cultured with 3T3-L1 adipocytes vs 3T3-L1 preadipocytes at +24 h. Differentially transcribed genes (FDR *q*-value<0.05) are highlighted in red for each indicated log_2_ FC range. Dashed red lines represent log_2_ FC thresholds −1 and 1. Data derived from RNAseq of three replicates/condition. **d** GSEA comparing RNAseq-generated global transcriptomes of 3T3-L1 adipocyte vs 3T3-L1 preadipocyte cultured Nalm-6 cells by KEGG pathway annotation. Significant pathways defined by FDR q-value <0.05. (Red bar: upregulated pathways, Green bar: downregulated pathways). **e** OP-Puro incorporation by Nalm-6 cells under 3T3-L1 preadipocyte (grey) or 3T3-L1 adipocytes (blue) coculture relative to monoculture. Nalm-6 monocultures treated with CHX, 10 µg/mL for 10 min (black), served as a positive control. Representative histograms of OP-Puro fluorescence are shown on the right. **f** OP-Puro incorporation in vitro in Nalm-6 cells in G0/G1 vs S/G2/M fractions +72 h after coculture with 3T3-L1 preadipocytes (grey) or 3T3-L1 adipocytes (blue). Data are normalized to monocultures. **g** OP-Puro incorporation in CD19+/CD45+ xenografted primary B-ALL cells (ALL24 and ALL25) harvested from femoral (blue) and tail (red) BMs according to cell cycle stage. ALL24 G0/G1 *p* = 0.0002, S/G2/M *p* = 0.0003; ALL25 G0/G1 *p* = 0.017, S/G2/M *p* = 0.045. **h** Western blot showing p-eIF2α levels in Nalm-6 cells (5×10^6^) after 72 h treatment with ISRIB, PERKi, or GCN2ib or Thapsigargin (250 nM), the latter serves as a positive control. (representative of two independent experiments). **i** OP-Puro incorporation in Nalm-6 cells cocultured with 3T3-L1 adipocytes after 72 h of GCN2ib (5ug/mL) vs vehicle treatment. The results are expressed relative to the OP-Puro fluorescence of Nalm-6 in 3T3-L1 preadipocyte coculture. **p = 0.0022. **j** Frequency of G0 cells in Nalm-6 cells cocultured with 3T3-L1 adipocytes after 72 h of GCN2ib (5ug/mL) vs vehicle treatment. **p = 0.0017. Data are presented as mean values ± SEM values of independent experiments (*n* = 2 in **c**, *n* = 3 in (**e**, **f**) assessing triplicates, *n* = 2 in (**g**) with *n* = 4 mice, *n* = 2 in (**i**, **j**) assessing triplicates. Statistical significance was assessed using 2-sided unpaired *t* tests (**e**, **f**, **g**, **i**, **j**) **p < 0.01. ***p < 0.001, ****p < 0.0001 or the precise *p*-value where indicated.

Initial assessments confirmed that a cell cycle checkpoint mechanism was not engaged in adipocyte-conditioned ALL cells (Supplementary Fig. 4a), suggesting that the phenotypic response of ALL cells to the adipocyte stroma likely occurs through an indirect vs a direct effect on the cell cycle. Next, given that lipid crosstalk is the principal mode of adipocyte-tumour cell interplay described to date^37–39^, we assessed the contribution of these events to the adipocyte-specific effects on ALL. Lipid profiling studies established that +24 h following 3T3-L1 adipocyte coculture, Nalm-6 cells demonstrated an accumulation of intracellular lipids, the components of which included oleic acid, a major subclass of fatty acid, which reflected, in part, a direct transfer of fatty acid from adipocytes to ALL, consistent with previous reports^37–39^. However, further functional evaluation of the fatty acid flux pathway confirmed that this was not a major factor driving ALL growth suppression (Supplementary Fig. 4b-j and Supplementary Discussion). Given that these candidate mechanisms could not robustly account for the adipocyte-ALL interplay, we next turned to unbiased screens. Here, we reasoned that the rapid adaptive response of ALL cells (Fig. 3b) suggests that global signalling and transcriptional networks were being stimulated. We therefore subjected Nalm-6 cells cocultured with 3T3-L1 adipocytes to quantitative LC-MS/MS phosphoproteomic analysis at two timepoints (+24 h and +72 h), comparing outcomes between 3T3-L1 preadipocyte coculture to control for general stromal effects. At both timepoints, we detected a substantial impact of adipocyte conditions on the ALL phosphoproteome (log_2_ FC ≥ 1 or log_2_ FC ≤ −1, *p*-value ≤ 0.05) involving 1,808 and 981 phosphorylation sites at +24 h and +72 h, respectively (Fig. 4a), consistent with large-scale rebalancing of these networks. Gene Ontology (GO) enrichment analysis was conducted on these significantly modulated phosphosites (Fig. 4b), revealing that at +24 h, terms related to biosynthetic and metabolic processes inclusive of lipid metabolism (e.g., lipid metabolic process, triglyceride catabolic process) were overrepresented, likely reflecting the ensuing effects of lipid translocation from 3T3-L1 adipocytes at this time (Supplementary Fig. 4b, c). In addition to these expected modulations, pathways relating to diverse aspects of protein homeostasis (protein translation, translation initiation, elongation “proteostasis”) and mRNA processing were significantly overrepresented in adipocyte-modulated ALL cells at both +24 h and +72 h, albeit switching from upregulation to repression over time. Notably, kinase-substrate enrichment analysis (KSEA), a platform to systematically infer the activation of given kinase pathways from mass spectrometry-based phosphoproteomics^40^, identified a general increase in proliferation-associated signalling networks, e.g., MAP2K1 JAK2, at +24 h despite robust suppression of cell growth (Supplementary Fig. 4k), indicating that mitogenic signalling was not acutely disrupted to explain the acute growth suppression.

Gene expression profiles of adipocyte-cultured Nalm-6 cells at +24 h assessed by RNAseq indicated predominantly *low-*magnitude changes (Fig. 4c, Supplementary Fig. 4l, m) with the difference in the adipocyte imprinted Nalm-6 transcriptome (adjusted *p*-value < 0.05) defined by only 547 genes, of which only a low number of genes (*n* = 50) met conventional DEG thresholds of log_2_ FC ≥ 1 or log_2_ FC ≤ −1.GSEA revealed few overrepresented pathways (Fig. 4d), namely, glycolysis (*p-*value < 0.05, FDR *q*-value=0.03) and its subcomponent fructose biosynthesis (*p-*value < 0.05, FDR *q*-value=0.03), likely reflecting the altered intracellular lipid environment (Supplementary Fig 4b, c). Notably, and in line with the phosphoprotetomics data, functions related to mRNA processing and multiple aspects of protein homeostasis (folding, amino acid metabolism, translation, ribosome biogenesis) were common pathways most underrepresented, as well as G2/M gene sets, as expected.

Together, these comparative results indicate that the acute response of ALL cells to adipocyte stimulation is characterized by significantly altered phosphorylation networks and, to a lesser extent, transcriptional events affecting cellular metabolism, a predicted outcome of ALL-adipocyte interaction, as well as major unanticipated effects on posttranscriptional processes and the protein translation network.

Given that protein homeostasis was a major cellular process modified by the adipocyte stroma, we next assessed its significance functionally by assessing its endpoint, global protein translation, using the O-propargyl-puromycin (OP-Puro) incorporation assay as a label of translational activity. The results demonstrated that compared to both control conditions, monoculture and 3T3-L1 preadipocytes, Nalm-6 ALL cells subjected to 3T3-L1 adipocyte coculture demonstrated strikingly lower OP-Puro incorporation, with very low technical and biological variability, achieving translational attenuation similar to that of cycloheximide (CHX), a robust inhibitor of protein translation (Fig. 4e). Similar outcomes were achieved in other ALL cell lines (Supplementary Fig. 4n), indicating that lowered rates of protein synthesis were not cell line specific. Importantly, differences in OP-Puro incorporation were not due to loss of fluorescence, which we confirmed occurred much later; 3 h after OP-puro administration (Supplementary Fig 4o). Moreover, there was no increased clearance of OP-puro containing polypeptides (Supplementary Fig. 4p) and no saturation in the OP-puro signal (Supplementary Fig. 4q). Furthermore, a similar loss of OP-Puro incorporation was also found in ALL cells cocultured with primary BM-MSC-derived adipocytes (Supplementary Fig. 4r), confirming that this was a relevant biological interaction not just restricted to the 3T3-L1 model system.

Next, to test whether differences in OP-puro are explained by differences in the cell cycle, we assessed OP-puro according to DNA content. We found that 3T3-L1 adipocytes still imposed significantly lower protein synthesis in ALL cells in both G0/G1 and S/G_2_/M (Fig. 4f and Supplementary Fig. 4s). Therefore, lower rates of protein synthesis in ALL cells were not simply a consequence of increased quiescence. Importantly, these findings were also consistent with analyses performed using human ALL samples in vivo. Two primary ALL tumours (ALL24 and ALL25) previously validated as growth repressive/dormancy responsive to in vivo adipocyte microenvironments (Fig. 3g, i) were subjected to repeated xenografting followed by ex vivo staining with OP-Puro. We found that primary ALL cells in vivo also demonstrated restricted protein translation in adipocyte-rich niches independent of the cell cycle stage (Fig. 4g), thus recapitulating the findings obtained with ALL cell lines in vitro; however, the effect was of lower magnitude, possibly due to some recovery of protein translation following a longer period of microenvironmental extraction.

Taken together, these data demonstrate that adipocyte microenvironments restrict the level of protein synthesis in ALL cells. Such reports are of particular interest because modulating protein synthesis is unique among prior reports of regulatory mechanisms involving adipocyte niches^31,37,39,41,42^. Furthermore, this observation directly raises the possibility that adipocyte-mediated cell cycle failure and ALL growth suppression reflect a limitation imposed by low proteome flux^43^.

To address this hypothesis, we first explored the mechanism of adipocyte-mediated hypotranslation using the 3T3-L1 adipocyte system. Assessment of general translation determinants established that there were no acute changes in cell diameter (Supplementary Fig. 4t) or compromise of transcriptional output (total RNA content, 18S or 28S ribosomal RNA) (Supplementary Fig. 4u) that explained the loss of translational output. Moreover, key signalling pathways representing major control points for protein translation, namely, mTOR^44^ and the endoplasmic reticulum stress-induced (ER) unfolded protein response plus correlated integrated stress response (ISR) pathways^45^, were not selectively engaged in ALL cells (Supplementary Fig. 4v) to explain the repression in protein translation. In line with this, there was no evidence of ER stress gene activation by either RNAseq (Fig. 4d) or RT-qPCR (Supplementary Fig. 4w). Thus, on the basis of these orthogonal assessments, 3T3-L1 adipocytes appear to modify ALL proteome flux via factors independent of canonical translation cascades, possibly suggesting that adipocytes execute their regulatory effects on protein translation via a distinct process.

To further establish whether ALL suppression is a consequence of adipocyte-mediated translational repression, we turned to pharmacological approaches that modulate the level of protein translation. Of drugs known to increase protein translation (ISRIB, PERKi, or GCN2ib), only GCN2ib led to effective target modulation (repressed eIF2α phosphorylation) when tested in Nalm-6 cells (Fig. 4h), and we therefore progressed this drug to testing in 3T3-L1 adipocyte coculture. We observed that the addition of GCN2ib led to a partial but notable increase in protein translation in ALL cells (Fig. 4i), which was associated with a significant reduction in adipocyte-associated ALL cell quiescence (Fig. 4j). Although GCN2ib treatment had no measureable impact on short-term growth, (Supplementary Fig. 4x), this likely reflects the incomplete rescue of protein translation induced by this treatment. Altogether, these data establish a causal association between lowered protein translation status in ALL cells and adipocyte-imposed ALL quiescence and underscore how subversion of a key housekeeping function contributes to the establishment of a therapy-adverse state.

### Adipocyte-adapted ALL proteomes increase global stress resistance

While adipocytes are known to contribute to chemoresistance via diverse mechanisms^14,17,19,22–27^, the link between ALL resistance and ALL hypotranslational states is least explored. In particular, given our finding that adipocyte-induced translational loss affects ALL tumours as a whole and involves all cell cycle stages (Fig. 4f-g), these data suggest that adipocyte niches may not exclusively protect leukaemia cells by maintaining them in the quiescent phase^24^.

**Fig. 5:**
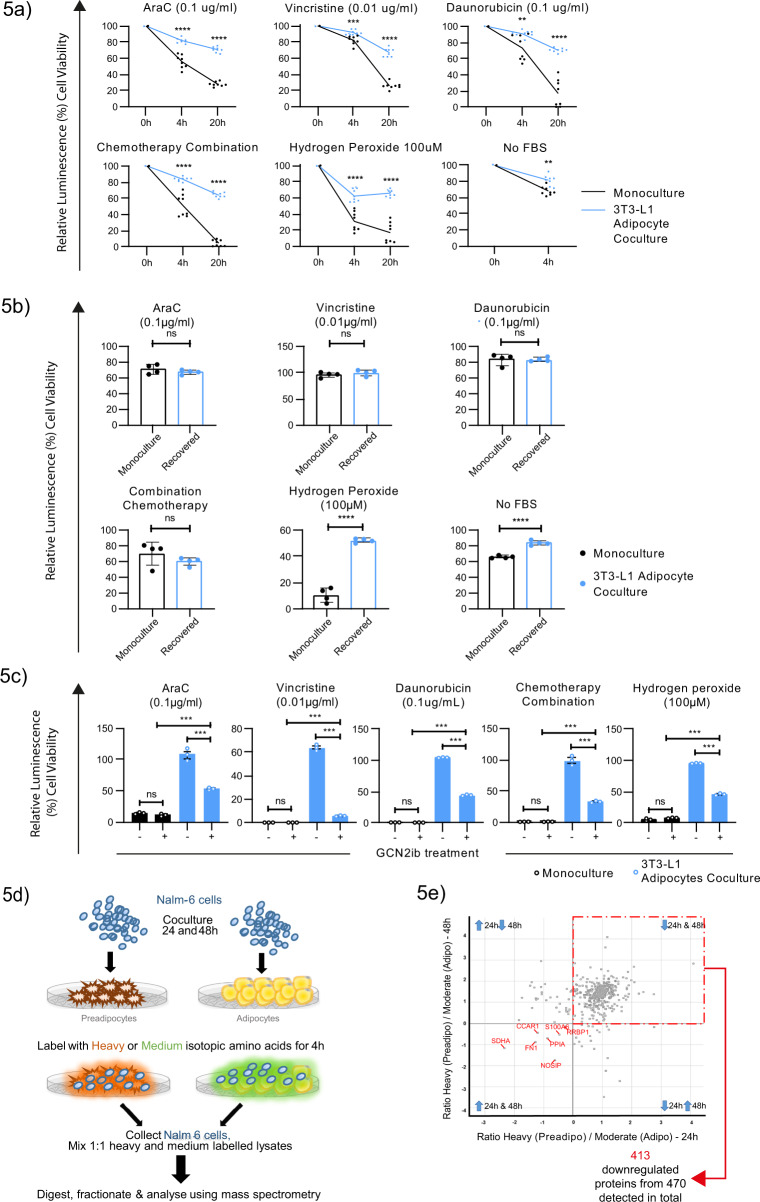
Adipocyte-adapted ALL proteomes increase global stress resistance. **a** Cell viability of Nalm-6 cells from monoculture (black, *n* = 8) vs 3T3-L1 adipocyte coculture (blue, *n* = 8) in the presence of the indicated chemotherapeutic agents or mitosis-independent stressors (no FBS and hydrogen peroxide). Data are presented relative to vehicle controls. **b** Cell viability of Nalm-6 cells after +48 h recovery from 3T3-L1 adipocyte coculture (blue, *n* = 4) or from monoculture (black, *n* = 4) after exposure to mitosis-dependant chemotherapy or mitosis-independent stressors (no FBS and hydrogen peroxide). Data are presented relative to vehicle controls. **c** Cell viability of Nalm-6 monocultured (black, *n* = 3) or 3T3-L1 adipocyte-cocultured Nalm-6 cells (blue, *n* = 3) in the presence of GCN2ib treatment (5 µg/mL) and the indicated cellular stressors. **d** Schematic for pulsed SILAC-based proteomic analysis. Nalm-6 cells cocultured with either 3T3-L1 adipocytes or preadipocytes were pulsed at +24 h and at 48 h with either ‘heavy’ or ‘medium’ isotope-labelled amino acids. Following a 4 h incubation, Nalm-6 cells were separated from their microenvironment for protein extraction. Lysates were mixed in equal amounts (between heavily and medium labelled samples at each time point), digested, fractionated and analysed using mass spectrometry.**e** Scatter plot showing the normalized log_2_ ratio of proteins detected following pulsed SILAC-based proteomic analysis in Nalm-6 cells cocultured with 3T3-L1 adipocytes (Adipo) relative to preadipocytes (preadipo) at +24 h vs 48 h from one experiment. Blue Arrows describe the direction of change in adipocyte-specific coculture. Data are presented as mean values ± SEM values of independent experiments (*n* = 2 in (**a**) with four replicates, *n* = 1 with four replicates in (**b**, **c**). Statistical significance was assessed using 2-sided unpaired *t* tests (**a**, **b** for the single-agent treatments), Mann–Whitney *U* test for combination chemotherapy treatment (**b**) One-way ANOVA followed by Tukey’s test for multiple comparisons **c**. **p < 0.01, ***p < 0.001, ****p < 0.0001.

To directly address whether adipocyte niches provide protective outcomes independent of target cell quiescence, we challenged Nalm-6 ALL cells with both cell cycle-dependent chemotherapeutics, an accepted instigator of quiescence-driven chemoresistance, and mitotic state-independent stresses induced by hydrogen peroxide or withdrawal of FBS. The latter would not be expected to drive differential outcomes based on target cell dormancy. Intriguingly, despite significantly reduced proteome flux, adipocyte-cultured ALL cells had increased cellular fitness against both cell cycle-dependent chemotherapy and mitotic state-independent stressors, although as expected, the outcomes were dependent on the individual stressors involved (Fig. 5a). Thus, adipocytes instigate more generalized cellular fitness in ALL cells beyond induction of cellular quiescence, which may relate to their global effect on protein translation.

In support of this notion, ALL cells were resensitized to chemotherapy following prolonged (+48 h) microenvironmental extraction (Fig. 5b), when protein biosynthetic activity and quiescence levels had normalized (Supplementary Fig. 5b) although continued to maintain a degree of adipocyte-imprinted resistance to mitotic state-independent stressors. Given the  possibility to pharmacologically modulate the adipocyte-imposed restrictions on the ALL proteome using GCN2ib (Fig. 4i) this offered the opportunity to directly test its putative contribution to cellular fitness. Therefore, we used GCN2ib to rescue protein translation in coculture prior to applying various cellular stresses. We observed that under GCN2ib treatment, the cytoprotective effect of adipocytes on ALL cells was decreased in response to cell cycle-dependent chemotherapy, as anticipated (Fig. 4j); however, we also observed a notable reduction in cytoprotection against mitotic state-independent stress 49-65% (mean ± SEM: 57% ± 2.4) (Fig. 5c). These observations indicate that adipocytes likely enable the survival of ALL cells via 2 distinct processes: induction of cellular quiescence and its wider suppression of protein translation.

Finally, we investigated the possibility that adipocyte-mediated stress protection is accompanied by activation of selective mRNA translation of proteins required for stress mitigation^45^. To investigate this hypothesis, we subjected Nalm-6 cells under 3T3-L1 adipocyte coculture to pulsed stable isotope chromatography-mass spectrometry (SILAC)-based proteomic analysis (Fig. 5d). As expected, we confirmed potent repression of global protein synthesis (Fig. 5e). Of the 470 proteins identified, 413 (87.5%) were robustly cross-identified as focally downregulated at both +24 h and +48 h. These co-downregulated proteins were enriched for pathways related to translational regulation, ribosomal assembly and mRNA processing (Table 1), reflecting widespread deregulation  of the translational network. Notably, we did not detect a high abundance of proteins subject  to selective mRNA translation (*n* = 7), suggesting that preferential mRNA translation was not actively engaged. Thus, we conclude that adipocyte-induced translational reprogramming accompanies a pro‐survival response that does not appear to be driven by a switch to a specialized translational programme.

**Table 1:** Pathway analysis of the co-downregulated proteins (n = 413) at +24 and +48 h in Nalm-6 cells cocultured with 3T3-L1 adipocytes, as detected by pulsed SILAC. Pathway enrichment was assessed by two-sided Fisher’s exact tests with a Benjamini–Hochberg FDR of 5%. Pathways with an enrichment >2 are presented.

	Category	Pathway	Enrichment factor	*P*-value	Benj. Hoch. FDR
1	Keywords	Elongation factor	3.1071	0.00034546	0.01708
2	GOCC	chaperonin-containing T-complex	3.1071	0.0001099	0.003187
3	Keywords	rRNA-binding	2.7618	0.00067496	0.026323
4	GOCC	cytosolic small ribosomal subunit	2.3052	1.25E−06	6.84E−05
5	GOCC	small ribosomal subunit	2.2597	1.31E−06	6.84E−05
6	GOBP	positive regulation of translation	2.244	0.00044973	0.036325
7	GOBP	regulation of cysteine-type endopeptidase activity	2.1749	0.00044019	0.036147
8	KEGG	Ribosome	2.1128	4.68E−11	1.04E−08
9	GOBP	nuclear-transcribed mRNA catabolic process, nonsense-mediated decay	2.0963	7.60E−12	7.49E−09
10	GOBP	nuclear-transcribed mRNA catabolic process	2.0714	1.11E−12	2.73E−09
11	GOBP	mRNA catabolic process	2.0605	1.02E−12	2.73E−09
12	Keywords	Citrullination	2.0315	0.00035833	0.01708
13	GOCC	cytosolic large ribosomal subunit	2.0232	5.17E−06	0.00018405
14	GOCC	cell cortex part	2.0196	0.0019023	0.045137
15	GOBP	RNA catabolic process	2.0196	1.73E−12	2.83E−09

## Discussion

Our study highlights several important findings. First, the adipocyte niche is dynamic in ALL, evolving from depleted states in ALL disease to full reconstitution during the remission response, confirming that these niches contribute temporally to an MRD-associated environment. Second, we identified a protein-based interplay regulated by the adipocyte stroma that restricts protein translation in ALL cells. Importantly, adipocyte-imposed restriction of protein translation played a direct role in regulating ALL cell quiescence. To the best of our knowledge, this is the first report to link the regulation of ALL quiescence with extrinsically mediated control of protein synthesis, although a similar parallel has been reported for HSCs involving exogenous angiopoietin^46^. Consistent with our findings, the level of translational activity has also been shown to regulate HSC maintenance^47^ further emphasizing the connection between quiescence-proliferation decisions and protein dynamics. We further show that reduced levels of global protein synthesis are associated with increased cellular fitness in ALL cells (Figs. 5a–c) and that this protection was not limited to the feature of cellular quiescence but encompassed broader multitrait resistance. In this way, adipocytes act not as selective chemo-survival factors but as providers of broader tumour cell protection than previously envisaged^48^. Notably, our finding that adipocytes enhance the survival and persistence of ALL cells through non-cell-autonomous suppression of the ALL proteome resonates with a form of biosynthetic stress resilience that has recently gained recognition as a cell-intrinsically determined process^49^, emphasizing the importance of attenuated biosynthetics as a critical mark of treatment resistance. How adipocyte-imposed hypotranslation leads to improved multistress resistance in ALL cells was not attributable to a switch to selective translation of prosurvival proteins, as reported for other protein suppression states^44,45^. This opens up the conceptual possibility that other factors, such as enhanced proteome quality, may play a role, analogous to paradigms in other model organisms^50^. Although these aspects are yet to be resolved, the observation that adipocyte-regulated loss of protein translation in ALL cells promotes ALL resistance adds to the increasing repertoire of stromal mechanisms that present a challenge to therapeutic success. In vivo targeting of adipocytes should provide further insight into this process but was not applied here due to the inability to modulate BM adipocytes exclusively without introducing significant confounding variables^51–53^. Therefore, appropriate model development will be essential to overcome this limitation and to establish the full in vivo potential of adipocytes in ALL disease/resistance.

Although our results implicate GCN2 as the potential factor determining ALL translational repression, given its rescue when GCN2 is inhibited (Fig. 4i), the lack of any selective increase in eIF2α phosphorylation under adipocyte conditions (Supplementary Fig. 4v) together with no orthogonal features of activated ISR downstream (Fig. 4d and Supplementary Fig. 4v-w) as well as incomplete phenotype reversal despite potent eIF2α inhibition would argue against this factor being the principal driver. We predict that the mechanism coupling adipocytes with the leukaemia proteome is complex and beyond the scope of this report given that canonical pathways controlling protein translation were not obvious. Significantly, our data do yield important insights into potential entry points for therapeutic intervention. First, therapeutic modulation of GCN2^54^ may disrupt the regulatory impact of adipocytes on ALL protein synthesis, thereby limiting the development of ALL cell phenotypes specifically linked to this process. Second, as adipocytes restrict the ALL proteome through direct interaction with ALL cells (Supplementary Fig. 5c, d), the latter showing importance in scenarios of clinical chemoresistance (Fig. 1), this suggests that physical interactions between ALL cells and adipocytes should be targeted to limit phenotypic plasticity arising from this specific stromal crosstalk which otherwise serves to increase tumour cell fitness. Further work is therefore warranted to deeply characterize the adipocyte-ALL interactome to pursue this potentially profitable avenue. The tractability of such approaches is supported by our data, which demonstrates that ALLproliferation loss,  lowered proteome flux and chemoprotection are  reversible states upon microenvironmental withdrawal (Fig. 5b and Supplementary Fig. 5d).

Our results merit comparison with the wider published data relating to adipocyte niches and leukaemia. While our data are in broad agreement with reports demonstrating that adipocyte stromal microenvironments are active modulators of the tumoural phenotype, the tremendous potential for these stroma to elicit both tumour- and tissue-dependent outcomes is also specifically highlighted.

**Fig. 6:**
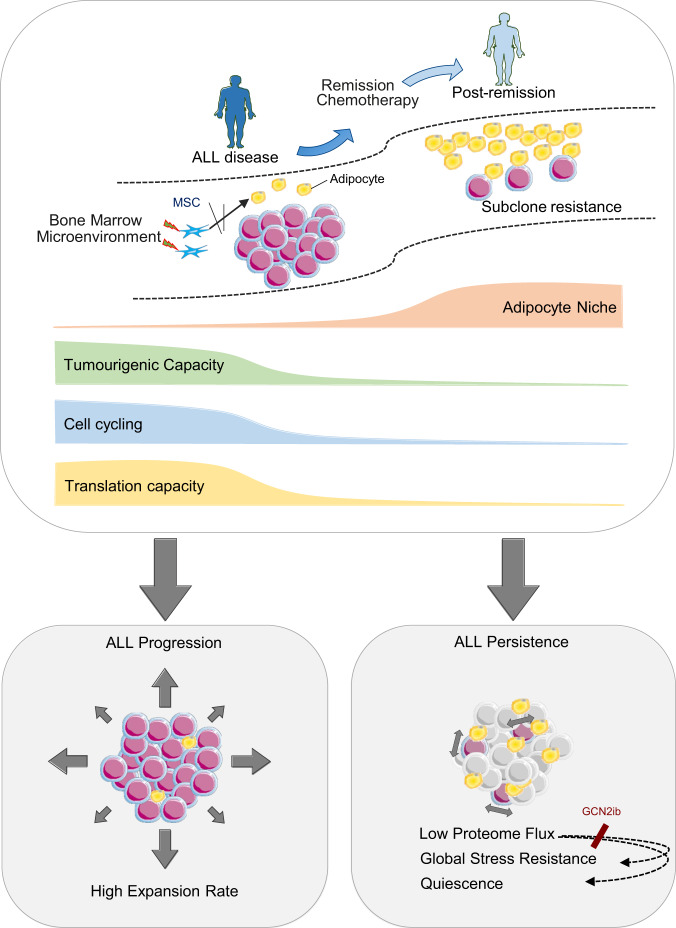
a Adipocyte niche function in ALL disease. Graphical abstract showing the temporal course of adipocyte niches across the ALL disease-remission transition and its pathophysiological relevance based on the demonstration of adipocyte-driven modulations in ALL cell phenotype.

In AML, where specifically evaluated, the adipocyte stroma has been shown to enhance AML proliferation as well as cellular survival^37^, similar to reported effects in a variety  of solid cancers^38,55,56^. In line with this, adipocytes located in gonadal tissue play a supportive role in CD36 ^+ ^AML leukaemia stem cell (LSC) maintenance and expansion^39^. The main mechanism defining the adipocyte-AML interplay relates to facilitated fatty acid transfer^37,39^. As our descriptions appear diametrically opposite, both in terms of the effect of adipocytes on cellular growth, the role of fatty acids and the nature of the modulation, which is a contextual and reversible property of interactions with adipocyte niches, this supports the notion of lineage specificity in adipocyte niche function. Thus, while adipocyte depletion states are characteristic of AML disease^31,57^ as well as other conditions, its biological significance will be defined by the interplay specific to the tumour cell type. Our study defines a conceptual framework (Fig. 6) specific to the B-ALL disease-remission transition, based on the interplay demonstrated with adipocyte stroma and should not be directly extrapolated to other conditions exhibiting BM-adipocyte dynamics.

Overall, our report provides insights into the versatility of adipocytes in their capacity for tumour adaptation. The functional coupling between adipocytes and ALL proteomes goes beyond known players of adipocyte-tumour cell interplay and represents an important mechanism of niche-driven resistance that should be targeted to increase therapeutic success.

## Methods

### Human samples

The use of human samples, collection and publication of individual-level clinical data was approved by the NRES Committee London; City & East Research Ethics Committee 10/H0704/65. The conduct of the study was fully compliant with the ethical approval. Pretreatment bone marrow and peripheral blood samples containing >80% blasts were collected from adult patients with ALL at diagnosis and cryopreserved after mononuclear cell (MNCs) isolation using Lymphoprep™ (07851, STEMCELL Technologies) based density gradient centrifugation. Patient- and disease-specific features are summarized in Supplementary Table 3. Adult healthy control BM-MNCs were purchased from Lonza (2M-125C) (median age: 27 years, range: 21-47). BM trephine biopsies were obtained from the posterior iliac crests of patients as part of routine diagnostic procedures and were sourced from the diagnostic laboratories of St Bartholomew’s, Royal Marsden and Christie Hospitals U.K. Healthy trephines were selected from adult lymphoma staging biopsies where histological involvement had been excluded. Peripheral blood stem cells were obtained from GCSF-mobilized healthy donors (GMPB) who were undergoing an allogeneic stem cell donation procedure on the COBE Spectra apheresis system (Caridian BCT, Lakewood, CA).

### Histological analysis

Quantitative analysis of adiposity in patient diagnosis, remission (<5% marrow blasts defined morphologically) and healthy control trephine biopsies was determined using a custom analysis protocol package (APP) designed using AuthorTM (Visiopharm). In brief, formaldehyde-fixed paraffin-embedded sections of human trephine biopsies were stained with haematoxylin and eosin (H&E) to visualize the gross anatomy of BM tissue. Whole H&E slides were scanned using a Pannoramic 250 Flash at  × 20 magnification and then imported into Visiopharm software. Regions of interest (ROIs) consisting of the haematopoietic marrow space were delineated. Trabecular bone, areas of haemorrhage, processing or tissue damage artefacts or non-marrow adipocytes were excluded from analysis. ROIs were verified by expert histopathological review. Filters were applied to the input image combining median, standard deviation and multiplication of pixel neighbourhoods followed by threshold analysis to distinguish the empty adipocytes from the background tissue. Post-processing scripts based on minimum and maximum area and form factor further restricted measurement to only adipocytes. A separation script was applied to distinguish individual adipocytes arranged in clusters. Output variables from the APP were adipocyte count and size along with the input area of the ROI. Immunostaining of BM trephines was performed on four μm slides for CD79a (clone SP18, Roche), CD34 (QBend 10, Roche), CD22 (clone SP104, Roche), Tdt (5267811001, Roche) and PAX5 (EPR3730, Abcam,). Staining was performed on the Ultra Ventena platform using the Optiview DAB detection kit (Roche 760700). Proximity relationships between drug resistant cells and adipocytes were determined by 2 independent assessors and verified by an expert haematopathologist. Only consensus events were reported.

### Adiponectin testing

BM serum samples were collected from patients with newly diagnosed ALL before the initiation of treatment. For control samples, BM serum was utilized from two sources: patients with < stage III diffuse large B cell lymphoma without BM involvement (*n* = 5) or healthy donors (*n* = 2). Adiponectin measurements were performed in technical duplicate using the commercial RayBio® Human Adiponectin ELISA kit (ELH-Arcp30).

### Generation of mesenchymal stromal cells

BM-MNCs were seeded at densities of 10 × 10^6^ cells per 175 cm2 in MesenCult™ MSC Basal Medium (05401, STEMCELL Technologies) supplemented with MesenCult™ MSC Stimulatory Supplement (05402, STEMCELL Technologies), 100 units/ml penicillin and 100 µg/ml streptomycin (15140122, Thermo Fisher Scientific), 2 mM l-glutamine and 1 ng/ml recombinant human FGF (233-FB, R&D Systems) and incubated at 37 °C and 5% CO_2_ under humidified conditions for 21 days. The culture medium was changed on day 2 and every week thereafter to remove nonadherent cells. On day 21, adherent cells, corresponding to BM-MSC populations at P1, were detached using a Trypsin/EDTA Solution (R001100, Thermo Fisher Scientific) and counted using the LUNA-FL™ Dual Fluorescence Cell Counter (Logos Biosystems) and Acridine Orange/Propidium Iodide Staining (F23001, Logos Biosystems). A subset was subjected to phenotypic characterization using the Human MSC Analysis Kit (562245, BD Biosciences) to ascertain whether the criteria defined by the International Society for Cellular Therapy were met: ≥95% expression of CD73, CD90 and CD105 and ≤2% expression of the haematopoietic and endothelial cell markers CD34, CD45, CD11b, CD19 and HLA-DR^30^. The rest of the cells were passaged to a maximum of P4.

### Colony-forming unit fibroblast (CFU-F) assays

For CFU-F assays, BM-MNCs were plated in duplicate or triplicate at densities of 1 × 10^6^ cells per 25 cm^2^ flask and cultured as described under “Generation of Mesenchymal Stromal Cells”. After 10 days, colonies (>50 cells) corresponding to CFU-F were stained with 0.5% crystal violet (C6158, Merck KGaA) and counted by light microscopy^58^.

### MSC differentiation

The ability of MSCs to differentiate into multiple mesenchymal lineages was examined using the Human Mesenchymal Stem Cell Functional Identification Kit (SC006, R&D Systems). All differentiation studies were undertaken at the P2-P3 stage, and MSCs were cultured in MEM α medium (22561021, Thermo Fisher Scientific) supplemented with 10% heat-inactivated foetal bovine serum (FBS; 10500064, Thermo Fisher Scientific), 100 units/ml penicillin, 100 µg/ml streptomycin and 2 mM l-glutamine prior to inducing differentiation. Adipogenic differentiation was initiated in confluent MSC cultures by adding adipogenic differentiation medium containing 1% adipogenic supplement. For osteogenic differentiation, MSCs were grown to 60–70% confluence prior to initiating osteogenic differentiation using osteogenic differentiation medium containing 5% osteogenic supplement. The culture medium was refreshed every 3 days for a total duration of 21 days, after which differentiated MSC cultures were set up for coculture. For immunofluorescence studies, MSCs were differentiated on sterile glass coverslips. For osteogenic differentiation, coverslips were coated with 1 µg/ml fibronectin (1918-FN, R&D Systems) to prevent cell detachment. Differentiated cells were fixed with 4% PFA for 20 min followed by washes in DPBS (no calcium, no magnesium; 14190094, Thermo Fisher Scientific). Cells were permeabilized and blocked with 0.1% Triton X-100 and 2% BSA (A9647, Merck KGaA) in DPBS for 45 min prior to incubation with either an unconjugated goat polyclonal antibody recognizing FABP4 (10 μg/mL; AF1443, R&D Systems) followed by a donkey anti-goat antibody conjugated to Alexa-647 (1:500; A-21447, Thermo Fisher Scientific) or an unconjugated mouse monoclonal antibody against osteocalcin (10 μg/mL; MAB1419, R&D Systems) followed by a goat anti-mouse antibody conjugated to Alexa-488 (1:500; A-11001, Thermo Fisher Scientific) to identify adipocytes and osteoblast cells, respectively. Slides were mounted with ProLong™ Gold Antifade Mountant with DAPI (P36931, Thermo Fisher Scientific), and fluorescent images were acquired at ×20 magnification using a Nikon Eclipse Ci fluorescence microscope. Images were analysed with ImageJ software (v1.50b, USA). At least five independent fields (>50 cells) per differentiation condition were used to quantify the frequency of DAPI/FABP4-stained adipocytes or DAPI/osteocalcin-stained osteoblasts.

### Conditioned medium (CM) collection

ALL cell lines (Nalm-6, REH, RS4;11) and three independent healthy human GCSF-mobilized peripheral blood (GMPB) samples enriched for haematopoietic stem cells were cultured in RPMI and 10% FBS for +72 h prior to supernatant collection. The cell viabilities prior to CM collection were >85%. Supernatants were filtered and centrifuged to eliminate cells and cell debris. Each CM was then aliquoted in a volume 5 ml with the addition of 50 µl of adipogenic supplement before being frozen at −80 °C prior to use. No CM controls were derived from RPMI, and 10% FBS cultured alone at +72 h was treated similarly. Precollected CM and non-CM controls were then utilized to induce adipogenic differentiation in P3 healthy MSCs, with medium changes as detailed under “MSC differentiation”.

### Oil red O staining

Oil red staining was performed by fixing cells with 4% PFA and then incubating them with 60% isopropanol for 5 min prior to staining with 0.3% oil red O (O0625, Sigma-Aldrich) in 60% isopropanol for 10 min at room temperature (RT). Cells were washed with distilled water to remove excess dye and counterstained with Mayer’s haematoxylin. Digital images were acquired using a Nikon Eclipse Ci microscope. To quantify the staining intensity, oil red was eluted in isopropanol, and absorbance was measured at a wavelength of 500 nm using a FLUOstar Omega (BMG Labtech) microplate reader.

### RNA sequencing

Total RNA was extracted from P4 ALL and healthy BM-MSCs as well as Nalm-6 cells cocultured with 3T3-L1 preadipocytes or 3T3-L1 adipocytes using the RNeasy mini kit (74104, Qiagen). RNA-seq was performed using NextSeq™ 500 High Output Run (150 cycles) with 40-50 million reads and 150 bp reads paired end. RNA-seq was performed in one technical run for the BM-MSC samples and in two technical runs for the Nalm-6 cells. RNA-Seq reads were aligned to the human genome (hg38) using Hisat2. Count files were generated by mapping reads to the human genome (hg38) p5 using HTSeq. Differential expression analysis was performed using Limma for BM-MSC RNA-seq and DESeq2 in R for Nalm-6 cell RNA-seq. The *p*-values were further adjusted using the Benjamini and Hocheberg procedure. Gene set enrichment analysis (GSEA) was performed using GSEA software version 2.1.0 (Broad Institute) and the FGSEA package in R (Broad Institute). Comparisons were made using curated (C2) gene set collections from the Molecular Signatures Database (MSigDB). The Ggplot2 package in R was applied to visualize the results. Publicly available gene expression datasets were used to compare gene expression profiles between mature human osteoblasts and adipocytes (GSE945129). Heat map generation was performed using individual probe sets. The array data have been deposited in the Gene Expression Omnibus repository (GEO accession number GSE151802).

### Cell lines

The human ALL cell lines Nalm-6 (CRL-3273), REH (CRL-8286) and RS4;11 (CRL-1873) were purchased from ATCC (American Type Culture Collection, Rockville, MD). ALL cell lines were cultured in RPMI 1640 medium (2187534, Thermo Fisher Scientific) supplemented with 10% FBS, 100 units/ml penicillin and 100 µg/ml streptomycin.

The murine fibroblast cell line 3T3-L1 (CL-173) was purchased from ATCC. 3T3-L1 cells were cultured in DMEM (41966029, Thermo Fisher Scientific) supplemented with 10% FBS, 100 units/ml penicillin and 100 µg/ml streptomycin.

All cells were incubated at 37 °C and 5% CO_2_ under humidified conditions and were routinely subjected to mycoplasma testing. Cell counts and viability measurements were performed with the LUNA-FL™ Dual Fluorescence Cell Counter (Logos Biosystems) using acridine orange/propidium iodide stain (F23001, Logos Biosystems).

### 3T3-L1 adipocyte differentiation

3T3-L1 preadipocytes were differentiated into adipocytes according to standard methods with slight modifications^59,60^. Briefly, two days post confluency (Day 0), DMEM (containing 10% FBS and antibiotics) was supplemented with 0.5 mM 3-isobutyl-1-methylxanthine (I7018, Merck KGaA), 0.25 μM dexamethasone (D2915, Merck KGaA), 2 μM rosiglitazone (R2408, Merck KGaA) and 1 μg/ml insulin (I9278, Merck KGaA). After 48 h (Day 2), the medium was replaced with DMEM supplemented with 10% FBS, 1 μg/ml insulin and antibiotics. The medium was subsequently changed to DMEM supplemented with 10% FBS and antibiotics every 2 days until day 10, after which cells were utilized for coculture experiments. The adipocyte conversion at this time was reproducibly 100%, as demonstrated by adipocyte-specific FABP4 immunofluorescence staining (Supplementary Fig. 3a).

### ALL coculture experiments

Fully confluent stromal cultures were washed twice with DPBS to remove differentiation media prior to adding ALL cell line suspensions to RPMI medium containing 10% FBS and antibiotics. For 150 mm culture dishes, the seeding density was 10 × 10^6^ ALL cells in 30 ml media, and for 6-well plates, 0.5 × 10^6^ ALL cells were seeded in 5 ml medium. The ratio of stromal cells to ALL cells at the time of coculture was consistently 1:2. For assaying cell number and cell cycle status, the full contents of cocultures, including stromal elements, were collected by flushing the wells with DPBS prior to flow cytometry analysis. To isolate ALL cells for reculture or RNA and protein extraction, cocultures were subjected to light trypsinization with a 50% diluted trypsin/EDTA solution for 1 min, followed by repeated washing with DPBS and gentle pipetting to remove supernatant and avoid disturbance of the stromal monolayer. Purity assessment of these supernatants by flow cytometry confirmed that stromal-cell contamination was reproducibly <5%.

In specified experiments, coculture was performed in the presence of drugs added 2 h after the coculture was set up corresponding to when ALL cells were fully adherent to the adipocyte stroma. The added drugs were 20 µM SSO (sulfo-N-succinimidyl oleate sodium; SML2148, Merck KGaA), 40 µM etomoxir (E1905, Merck KGaA), 30 µM BMS309403 (5258, Tocris Bioscience) and GCN2ib (HY-112654, Generon).

### Flow cytometry

Flow cytometry was performed on an LSRFortessa™ flow cytometer (BD Biosciences). Data were analysed by FlowJo (version 10.1, Tree Star). Antibody staining was performed for 15 min in the dark. DAPI (D3571, Thermo Fisher Scientific, 1:2000 in DPBS containing 2% FBS) was added prior to flow cytometric analysis. Gates were set up to exclude doublets, nonviable cells (DAPI+, or fixable viability dye+) and isotype-stained populations. Cell counts were assessed using CountBright™ Absolute Counting Beads (C36950, Thermo Fisher Scientific) on CD19+-gated populations.

Apoptotic cell death was assessed by dual staining with Annexin V-Alexa Fluor 647 (A23204, Thermo Fisher Scientific) and DAPI in CD19+-gated populations. Cells were washed in Annexin V binding buffer (556454, BD Biosciences) and stained with the B lineage ALL marker CD19-PE (clone 4G7; 345777, BD Biosciences) and Annexin V Alexa Fluor 647 for 15 min, protected from light. DAPI (1:2000 in Annexin V binding buffer) was added prior to flow cytometric analysis.

Cell cycle assessment was performed by Ki67 and DAPI dual staining on CD19+-gated populations. Cells were washed in DPBS containing 2% FBS and stained with CD19-PE and Fixable Viability Dye eFluor™ 780 (65-0865-14, Thermo Fisher Scientific) for 30 min at 4 °C. Cells were then fixed with ice-cold 70% ethanol and kept at −20 °C. Subsequently, the samples were washed twice in DPBS containing 2% FBS, stained with Ki67-FITC (556026, BD Biosciences) for 30 min, and protected from light. DAPI (1:500 in DPBS containing 2% FBS) was added prior to flow cytometric analysis. Gating of the different phases of the cell cycle was determined by population analysis and isotype controls.

Intracellular lipid content was assessed by BODIPY™ 493/503 staining. Cells were washed in DPBS and stained with BODIPY™ 493/503 (4,4-Difluoro-1,3,5,7,8-Pentamethyl-4-Bora-3a,4a-Diaza-s-Indacene) (D3922, Thermo Fisher Scientific) for 30 min at 37 °C. Subsequently, the samples were washed in DPBS, and DAPI (1:2000 in DPBS) was added prior to flow cytometric analysis. BODIPY median fluorescence intensity (MFI) was determined as a quantification of intracellular lipid content.

For the assessment of the expression of the transmembrane fatty acid transporter CD36, cells were washed in DPBS containing 2% FBS and stained with CD36-APC (clone CB38; 550956, BD Biosciences) together with CD19-PE and CD10-APC (clone HI10a 332777 BD Biosciences) for primary ALL samples or CD34-APC (clone; 555824 BD Biosciences) together with CD33-PE (clone WM53; 555450, BD Biosciences) for AML samples. Gating strategies for flow cytometry are summarized in Supplementary Fig. 6.

### Osmium staining

Following euthanasia, murine tibiae were isolated, thoroughly cleaned and fixed in 10% formalin at 4 °C for 48 h. Bones were decalcified for 14 days in 14% EDTA and washed in Sorensen’s phosphate buffer. Bones were then stained for 48 h in 1% osmium tetroxide (Agar Scientific), washed in Sorensen’s phosphate buffer and embedded in 1% agarose, forming layers of five tibiae arranged in parallel in a 30 ml universal tube. Tubes of embedded tibiae were then mounted in a Skyscan 1172 desktop micro-CT (Bruker microCT, Kontich). Samples were scanned through 360° using a step of 0.40° between exposures. A voxel resolution of 12.05 μm was obtained in the scans using the following control settings: 54 kV source voltage, 185 μA source current with an exposure time of 885 ms. A 0.5 mm aluminium filter and two-frame averaging were used to optimize the scan. After scanning, the data were reconstructed using NRecon v1.6.9.4 software (Bruker, Kontich, Belgium). The reconstruction thresholding window was optimized to encapsulate the target image. Volumetric analysis was performed using a CT Analyser v1.13.5.1 (Bruker microCT, Kontich).

### Xenotransplantation experiments

All animal experiments were performed under license PPL 70/8540 approved by the Home Office of the United Kingdom and in accordance with institutional guidelines. Immunodeficient male (to avoid gender-based variation in adiposity) NSG mice (NOD. Cg-Prkdcscid 112rgtm1Wjl/SzJ) were obtained from Charles Rivers Laboratories. All mice were housed in barrier accommodation in the Biological Services Unit at Queen Mary University of London, Charterhouse Square. Thawed MNCs obtained from PB or BM from ALL patients at diagnosis were resuspended in DPBS containing 10% FBS, and 5-8 × 10^6^ cells were administered intravenously into the tail vein of 7- to 10-week-old nonirradiated mice. For Nalm-6 xenotransplantation, 0.5 × 10^6^ cells were injected. Engraftment of human leukaemic cells was examined every two weeks by intratibial bone marrow sampling under anaesthesia (isoflurane) and postprocedure analgesia (0.1 mg/kg vetergesic). Animal wellbeing and weight were regularly monitored, and mice were euthanized when signs of disease-related symptoms developed, in compliance with approved protocols or at leukaemia cell engraftment of >70%, as determined by intratibial sampling. This threshold was approximately reached 6–8 weeks after tumour inoculation of primary ALL and 3-4 weeks with the Nalm-6 ALL cell line. At sacrifice, bone marrow cells from the femur and the tail vertebrae were isolated by mechanically crushing the bones with a mortar and pestle in DPBS containing 2% FBS, followed by filtration through a 40 µM nylon mesh to obtain a single-cell suspension. Following centrifugation, cell pellets were subjected to red blood cell lysis using ammonium chloride (07850, STEMCELL Technologies) prior to flow cytometric analysis. Gonadal fat tissue was microdissected at sacrifice, chopped and then digested with Liberase (5401119001, Roche) for 30 min at 37 °C and processed as previously described^39^. Flow cytometric assessment of primary human leukaemia engraftment was performed by CD19-PE and CD45-FITC (clone 2D1; 345808, BD Biosciences) costaining of DAPI-viable cells or CD19-PE and CD10-FITC (clone W8E7; 347503, BD Biosciences) costaining in the case of Nalm-6 engraftment. Cell cycle analysis was performed on CD19 + -gated populations as described under “Flow cytometry”. For Op-Puro analysis, 0.5 × 10^6^ cells from femoral or tail bone marrow were incubated with 10 µM OP-Puro reagent in RPMI medium containing 10% FBS for 20 min at 37 °C. OP-Puro incorporation analysis was performed on CD19- and CD45 + -gated populations as described under “O-propargyl-puromycin (OPP) labelling”.

### Western blot analysis

Total cellular protein content was extracted in NuPAGE™ LDS Sample Buffer (NP0007, Thermo Fisher Scientific) containing 50 mM dithiothreitol (NuPAGE™ Sample Reducing Agent; NP0009, Thermo Fisher Scientific). Protein content was quantified using the Pierce™ BCA Protein Assay Kit (23227, Thermo Fisher Scientific). Proteins were separated on NuPAGE™ 4–12% Bis–Tris polyacrylamide gels (NP0335BOX, Thermo Fisher Scientific) and transferred onto 0.45 µM PVDF transfer membranes (88518, Thermo Fisher Scientific). Membranes were blocked in Tris-buffered saline containing 0.1% TWEEN 20 and 5% BSA for 60 min at RT and then incubated with primary antibodies overnight at 4 °C. HRP-conjugated secondary antibodies were used at a 1:5000 dilution (anti-rabbit 7074 and anti-mouse 7076, Cell Signaling Technology). Protein signals were developed using SuperSignal™ West Pico PLUS Chemiluminescent Substrate (34577, Thermo Fisher Scientific), and images were acquired using an Amersham Imager 600 RGB (GE Healthcare). Densitometric analysis of immunoblots was performed using ImageJ software.

Primary antibodies were purchased from Cell Signaling Technology: P21 (12D1, 2947), P27 (D69C12, 3686), phospho-P53 (Ser46, 2521), Cyclin D1 (E3P5S, 55506), Cyclin E (HE12, 4129), phospho-Rb (Ser807/811, 8516), E2F-1 (3742), Phospho-AMPK (Thr172, 2531), Phospho-mTOR (Ser2448; 2971), Phospho-S6 Ribosomal Protein (Ser235/236; 2211), S6 Ribosomal Protein (5G10; 2217), Phospho-4EBP1 (Thr37/46; 2855), 4EBP1 (53H11; 9644), Phospho-eIF2α (Ser51; 9721), eIF2α (9722) and ATF4 (D4B8; 11815). The anti α-Tubulin antibody was purchased from Abcam (DM1A; ab7291). All primary antibodies were diluted 1:1000. In specified experiments, ISRIB (200 nM, S7400, Selleckchem), PERK inhibitor (2 µM; GSK2606414) or GCN2ib (5 µg/mL; HY-112654), 250 nM thapsigargin (1138, Tocris Bioscience) or 1 µM Torin 1 (4247, Tocris Bioscience) prior to cell harvest and lysate collection.

### Lipidomics

Two technical replicates of ALL cells (3 × 10^6^) were harvested from 3T3-L1 adipocytes or preadipocytes as described under “ALL coculture experiments”. Cell pellets were washed twice in ice-cold DPBS, prepared by snap freezing on dry ice and submitted to MS-Omics for lipidomic profiling. Lipids were extracted from the samples using methanol, ultrapure water and methyl tert-butyl ether. The organic phase used for lipid analysis was separated, transferred to injection vials, dried under nitrogen flow and reconstituted in an isopropanol/acetontril/water mixture. For quality control, a mixed pooled sample (QC sample) was created by taking a small aliquot from each sample. This sample was analysed at regular intervals throughout the sequence. Matrix effects were tested for quantified compounds by spiking the QC sample at a minimum of two levels. Chromatographic separation was performed using a UPLC system (Vanquish, Thermo Fisher Scientific) coupled with a high-resolution quadrupole-orbitrap mass spectrometer (Q Exactive™ HF Hybrid Quadrupole-Orbitrap, Thermo Fisher Scientific). An electrospray ionization interface was used as the ionization source. Analysis was performed in negative and positive ionization modes. A QC sample was analysed in MS/MS mode for identification of compounds. UPLC was performed using a slightly modified version of the protocol described by Isaas et al.^61^. Data were processed using Compound Discoverer 3.0 (Thermo Fisher Scientific).

### Oleic acid treatment

ALL cells at a concentration of 0.2 × 10^6^ cells in 1 ml RPMI medium (containing 10% FBS and antibiotics) were treated with 30 or 100 µM oleic acid-albumin from bovine serum (O3008, Merck KGaA) for 24, 48 or 72 h. Treatment with 0.3% bovine serum albumin (A7511, Merck KGaA) was used as a control. An additional 1 ml of medium with the corresponding treatment was supplemented after 24 h. Cell counts and viability measurements were performed using acridine orange/propidium iodide staining.

### Fatty acid translocation experiments using BODIPY

To examine directional lipid transfer from adipocytes to ALL cells, 3T3-L1 adipocytes were incubated with 2 µg/ml BODIPY™ 558/568 C_12_ (4,4-Difluoro-5-(2-Thienyl)-4-Bora-3a,4a-Diaza-s-Indacene-3-Dodecanoic Acid) (D3835, Thermo Fisher Scientific) for 4 h. The adipocytes were washed with HBSS containing 0.2% fatty acid-free BSA to remove extracellular fatty acids. Subsequently, ALL cell lines at a concentration of 0.2 × 10^6^ cells/ml were cocultured with labelled 3T3-L1 adipocytes for 24 h before being harvested, washed with HBSS containing 0.2% fatty acid-free BSA and analysed by an Amnis® ImageStream®XMk II flow cytometer. Imagining was performed using INSPIRE™ software.

### Short hairpin RNA CD36 targeting

Knockdown of CD36 was achieved using MISSION® shRNA lentiviral transduction particles cloned into the pLKO.1 lentiviral vector (CD36 shRNA: SHCLNV, nonmammalian shRNA control: SHC002V, Merck KGaA). Sequences of transduction particles are listed in Supplementary Table 4. Cells were plated in 6-well plates (0.25 × 10^6^ cells/well) and treated with 5 µg/ml polybrene for 15 min prior to transduction with the appropriate lentiviral particles at a multiplicity of infection of 1. Cells were incubated with the particles for 72 h, and then selection with 1.0 µg/ml puromycin was performed until use in experiments. Cells were assayed for knockdown of CD36 by RT-qPCR.

### Quantitative real-time PCR

RNA was extracted with a RNeasy Micro Kit (74004, Qiagen) following the manufacturer’s protocol. RNA was quantified (total RNA, 18S rRNA and 28S rRNA), and RNA integrity was assessed using an RNA ScreenTape (5067-5576, Agilent), RNA ScreenTape Sample Buffer (5067-5577, Agilent) and the 4200 TapeStation System (G2991AA, Agilent). RNA was reverse transcribed using the High-Capacity cDNA Reverse Transcription Kit (4368814, Thermo Fisher Scientific). Quantitative real-time PCR was performed on a Touch™ Real-Time thermal cycler (CFX384, Bio-Rad) using SsoAdvanced™ Universal SYBR® Green Supermix (1725271, Bio-Rad). Target primer sequences are listed in Supplementary Table 5. β-Actin was used to normalize the RNA content. All samples were analysed in triplicate and averaged.

### Phosphoproteomic LC-MS/MS analysis

Nalm-6 cells were cocultured with 3T3-L1 adipocytes or preadipocytes for 24 or 72 h in 150 mm culture dishes (see ALL coculture experiments). Cells were isolated from coculture, and cell lysis and trypsin digestion were performed following previously described protocols in urea buffer (8 M urea in 20 mM in HEPES pH 8.0 supplemented with 1 mM Na_3_VO_4_, 1 mM NaF, 1 mM Na_4_P_2_O_7_ and 1 mM sodium β-glycerophosphate)^40,62^. Briefly, proteins were quantified by BCA assay, and 250 µg of protein was reduced and alkylated by sequential incubation with 10 mM dithiothreitol and 16.6 mM iodoacetamyde. Urea was diluted to a concentration of 1.44 M with 20 mM HEPES (pH 8.0), and samples were incubated with trypsin beads [50% slurry of TLCK-trypsin (20230, Thermo-Fisher Scientific)] on a thermoshaker overnight. Peptide solutions were then desalted using 10 mg OASIS-HLB cartridges (Waters, Manchester, UK) and eluted with glycolic acid buffer (1 M glycolic acid, 50% ACN, 5% TFA). Phosphopeptides were enriched as previously described using 50 µL of TiO_2_ beads [(50% slurry in 1% TFA) GL Sciences]^40,62^. For phosphopeptide recovery, peptides were eluted in 5% NH_4_OH; dried peptide extracts were then dissolved in 0.1% TFA and analysed in an LC-MS/MS system. This consisted of a nanoflow ultra-high pressure liquid chromatography system nanoflow ultimate 3000 RSL nano (Dionex) coupled to a Q Exactive™ Plus Orbitrap™ Mass Spectrometer (Thermo Fisher Scientific).

The LC system used mobile phases A (3% ACN: 0.1% FA) and B (100% ACN; 0.1% FA). Peptides were loaded onto a μ-precolumn (160454) and separated in an analytical column (EASY-Spray; ES803). The gradient was 1% B for 5 min and 1% B to 35% B for 60 min. Following elution, the column was washed with 85% B for 7 min and equilibrated with 3% B for 7 min at a flow rate of 0.25 µL/min. Peptides were nebulized into the online connected Q-Exactive Plus system operating with a 2.1 s duty cycle. Acquisition of full-scan survey spectra (*m/z* 375–1500) with a 70,000 FWHM resolution was followed by data-dependent acquisition in which the 15 most intense ions were selected for HCD (higher energy collisional dissociation) and MS/MS scanning (200–2000 *m/z*) with a resolution of 17,500 FWHM. A 30 s dynamic exclusion period was enabled with an exclusion list with a 10 ppm mass window. The oxverall duty cycle generated chromatographic peaks of approximately 30 s at the base, which allowed the construction of extracted ion chromatograms (XICs) with at least ten data points.

### Peptide identification and quantification

Mascot Distiller 2.7.1 was used to fit an ideal isotopic distribution to the MS/MS data to maximize peptide identification, and the Mascot Daemon 2.6 search engine was used to match peaks to peptides in proteins present in the UniProt/SwissProt Database (human species). The process was automated with Mascot Daemon 2.5.0, mass tolerance was set to ± 10 ppm, and variable modifications phospho (ST), phospho (Y) gln→pyro-glu (N-term Q) and oxidation (M) were included in the search. Carbamidomethyl (C) as fixed modification. Trypsin was selected as the digestion enzyme, and 2 missed cleavages were allowed. Sites of modification were reported when they had delta scores >10. Peptide and subsequent protein quantification was achieved using in-house developed Peak statistics calculator (PESCAL) software^63^. PESCAL constructs XICs for each peptide identified with the MASCOT search engine. With each constructed XIC, peak heights could be calculated. These peptide peak heights were then normalized to the sum of the intensities for each individual sample, and the average fold change between conditions could be determined. Statistical significance between conditions was considered significant when Student’s *t* tests produced a *p*-value<0.05 following correction with Benjamini–Hochberg multiple testing. Further data processing and analysis were conducted within Microsoft Excel (2013) or R (v3.4.4-reshape2, ggplot2, gplots, readxl, Hmisc and ggrepel packages). The Z-score was calculated based on the fold change, as previously described^40,64^.

### KSEA and gene ontology analysis

Kinase-substrate enrichment analysis (KSEA) was performed as follows^40^; briefly, peptides differentially phosphorylated between a set of samples (at non-adjusted *p*-value<0.05) were grouped into substrate sets known to be phosphorylated by a specific kinase as annotated in the PhosphoSite, Phospho. ELM, and PhosphoPOINT databases^65–67^. To infer enrichment of substrate groups across sets of samples, the hypergeometric test was used, followed by correction with Benjamini–Hochberg multiple testing. For gene ontology analysis, proteins differentially phosphorylated between conditions (at non-adjusted *p*-value<0.05) were grouped into gene ontologies (biological process) as annotated in UniProt databases. To infer ontology enrichment across sets of samples, the hypergeometric test was used, followed by correction with Benjamini–Hochberg multiple testing.

### O-propargyl-puromycin (OPP) labelling

The Click-iT™ Plus O-propargyl-puromycin (OPP) Alexa Fluor™ 647 Protein Synthesis Assay Kit (C10458, Thermo Fisher Scientific) was used to measure global protein synthesis. ALL cells were isolated from their microenvironment as described under “ALL coculture experiments” and immediately incubated with 10 µM OP-Puro for 20 min at 37 °C in ALL cell line-specific media, RPMI containing 10% FBS and antibiotics. In specific experiments, 10 μg/ml cycloheximide (CHX; C4859, Merck KGaA) was added 10 min prior to OP-Puro to block protein translation, thereby serving as a positive control. Cells were subsequently washed in DPBS containing 2% FBS and stained with CD19-PE. A further wash was performed prior to fixation with the Fixation/Permeabilization Solution Kit (554714, BD Biosciences). Incubation with the Click-iT® Plus OPP reaction cocktail provided in the kit containing Alexa Fluor® 647 picolyl azide for 30 min led to the “click” reaction and azide-alkyne cycloaddition. Additional DPBS containing 2% FBS washes followed prior to addition of DAPI (1:2000 in DPBS containing 2% FBS) and flow cytometric analysis. OP-Puro MFI was measured to quantify protein synthesis. To assess the effect of proteasome activity on OP-Puro incorporation, cells were treated with 10 µM MG-132 (M7449, Merck KGaA) for 2 h. At the final 20 min of incubation, 10 µM OP-Puro reagent was added.

For the rescue experiment, ALL cells were cocultured with either 3T3-L1 adipocytes or preadipocytes for 24 h, and then 0.5 × 10^6^ cells were extracted from their microenvironment, washed and recultured in fresh RPMI medium for an additional 48 h. ALL cells (0.5 × 10^6^) were plated in parallel as a monoculture control. Cell number and OP-puro incorporation at 24 and 48 h following microenvironmental extraction were assessed.

### In vitro Transwell experiments

Transwell assays were performed as previously described^68^. In brief, 0.5 × 10^6^ Nalm-6 cells resuspended in 5 ml RPMI containing 10% FBS and antibiotics were added to the upper chamber, with confluent 3T3-L1 adipocytes or preadipocytes placed in the bottom chamber of a 24 mm Corning® Transwell system with 0.4 μm pore polyester membrane inserts (CLS3450, Corning NY Merck KGaA). After +24 h, the number of CD19 ^+ ^Nalm-6 cells in the upper chamber and OP-puro incorporation in CD19 ^+ ^Nalm-6 cells were quantified as described under “Flow cytometry” and “O-propargyl-puromycin (OPP) labelling”.

### Adipocyte secretome experiment

3T3-L1 adipocytes and preadipocytes were cultured for 72 h in 100% confluent 100 mm cell culture dishes, and culture medium was collected and stored at −20 °C until use. Nalm-6 cells were adapted to DMEM for 72 h prior to seeding at a concentration of 2 × 10^6^ cells in 4 ml adipocyte- or preadipocyte-conditioned medium, following which cell number (using CountBright™ Absolute Counting Beads) and protein translation were assessed as described under “Flow Cytometry” and “O-propargyl-puromycin (OPP) labelling”.

### Stress experiments

Nalm-6 cells were isolated from monoculture or from 3T3-L1 adipocyte coculture at 48 h, washed twice in DPBS and resuspended in ALL cell line-specific media at equivalent densities of 10^5^ cells/100 µl per well in black NuncTM F96 MicroWellTM plates (137101, Thermo Fisher Scientific). Subsequently, various treatments were added (final concentrations): 0.1 µg/ml cytarabine, 0.01 µg/ml vincristine, 0.1 µg/ml daunorubicin or a combination of the three chemotherapeutic agents with 100 µM hydrogen peroxide (H1009, Merck KGaA). Nutritional deprivation was induced by replating cells without the addition of FBS. In specified experiments, GCN2ib (5 µg/mL; HY-112654, Selleckchem) was added along with the indicated cellular stressor(s). At the indicated times, cell viability was assessed using the CellTiter-Glo® Luminescent Cell Viability Assay according to the manufacturers’ instructions. Luminescence was recorded using a FLUOstar Omega microplate reader. Luminescence signals were normalized to untreated controls at the respective timepoints. For confirmation, untreated controls were assessed at +4 h following microenvironmental extraction to confirm the persistence of translation arrest using O-propargyl-puromycin (OPP) labelling (**S5a**).

### Pulsed SILAC-based proteomic analysis

Nalm-6 cells were cocultured with 3T3-L1 adipocytes or preadipocytes for 24 or 48 h in 150 mm culture dishes (see ALL coculture experiments). Subsequently, the cells were pulsed with either ‘heavily’ (H) or ‘moderately’ (M) isotope-labelled amino acids for +4 h. Cells were then isolated from coculture, lysed in 2% SDS and 50 mM Tris-HCl pH 7.5, sonicated and balanced following protein quantification. Equal amounts of the corresponding H&M lysates were then mixed together and subsequently trypsin digested using the filter-aided sample preparation (FASP) method^69^. Pooled peptides were then fractionated into seven peptide fractions using the Pierce™ High pH Reversed-Phase Peptide Fractionation Kit (84868, Thermo Fisher Scientific) according to the manufacturers’ instructions. Different fractions were then lyophilized and resuspended in 0.1% TFA and 2% acetonitrile prior to analysis on a Q Exactive™ Plus Orbitrap™ Mass Spectrometer coupled with a nanoflow ultimate 3000 RSL nano HPLC platform (Thermo Fisher Scientific). Briefly, samples were resolved at a flow rate of 250 nl/min on an Easy-Spray 50 cm × 75 μm RSLC C18 column (Thermo Fisher Scientific) using a 123 min gradient of 3% to 35% of buffer B (0.1% formic acid in acetonitrile) against buffer A (0.1% formic acid in water), and the separated samples were infused into the mass spectrometer by electrospray. The spray voltage was set at 1.95 kV, and the capillary temperature was 255 °C. The mass spectrometer was operated in data-dependent positive mode with 1 MS scan followed by 15 MS/MS scans (top 15 methods). The scans were acquired in the mass analyser at 375–1500 *m/z* range, with a resolution of 70,000 for the MS scans and 17,500 for the MS/MS scans. Fragmented peaks were dynamically excluded for 30 sec. MaxQuant (version 1.6.3.3) software was used for the database search and SILAC quantification of mass spectrometry raw files^70^. The search was performed against a FASTA file of the human proteome extracted from UniProt.org. A precursor mass tolerance of 4.5 ppm and a fragment mass tolerance of 20 ppm were applied. Methionine oxidation and N-terminal acetylation were included as variable modifications, while carbamidomethylation was applied as a fixed modification. Two trypsin miscleavages were allowed, and the minimum peptide length was set to seven amino acids. All other MaxQuant parameters apart from the ratio count cut-off (set at 1) were kept as defaults. All raw files were searched together with the match between runs option set as enabled and re-quantify option as disabled. All downstream data analyses were performed with Perseus (version 1.5.5.3)^71^ using the MaxQuant ProteinGroups.txt output file. Briefly, protein H/M ratio values were converted to Log_2_ scale. Reverse (decoy) hits, potential contaminants, and proteins identified only by modified peptides were filtered out. Proteins were also filtered to have at least one valid H/M SILAC value. Fisher’s exact test was performed with Perseus using a Benjamini–Hochberg FDR of 5%.

### Statistical analysis

Data are presented as the mean with standard error of the mean or as the median with interquartile ranges. Results with a *p*-value<0.05 were considered statistically significant. Statistical analyses were performed using GraphPad Prism 8 software (GraphPad, San Diego, CA). All data were subjected to normality testing prior to statistical assessment with software-recommended tests.

### Reporting summary

Further information on research design is available in the Nature Research Reporting Summary linked to this article.

## Supplementary information

Supplementary Information

Reporting summary

## Data Availability

The datasets generated during the current study have been deposited to the Gene Expression Omnibus repository via GEO accession number GSE151802. All raw mass spectrometry files reported in this paper have been deposited to the ProteomeXchange Consortium via, PXD019186 and PXD019369. All source data supporting the findings of this study are available within the article and its supplementary information files and from the corresponding author upon reasonable request. Source data are provided with this paper.

## Source data

Source Data

## Code availability

Custom pipelines for BM-adipocyte analysis using Visiopharm software are available upon reasonable request to the corresponding author.
