# Supplementary material for: Adipocytes disrupt the translational programme of acute lymphoblastic leukaemia to favour tumour survival and persistence
Source: Nat Commun. 2021 Sep 17;12:5507. doi: 10.1038/s41467-021-25540-4 (PMC8448863; doi:10.1038/s41467-021-25540-4)
Supplement: Supplementary file 1 — Supplementary Information [file 41467_2021_25540_MOESM1_ESM.pdf]

Supplementary Information

# Adipocytes disrupt the translational programme of acute lymphoblastic leukaemia to favour tumour survival and persistence

Heydt et al.

## Supplementary figures

**a**

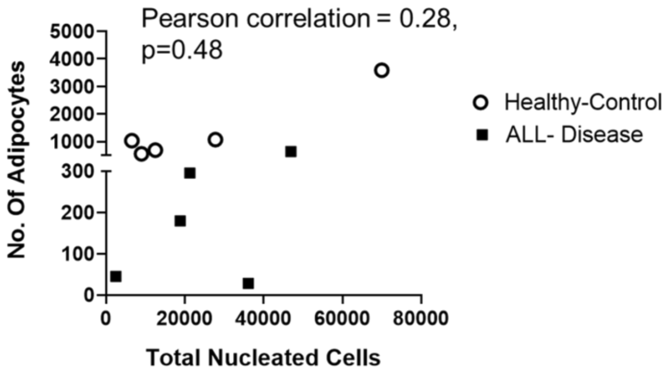


**b**

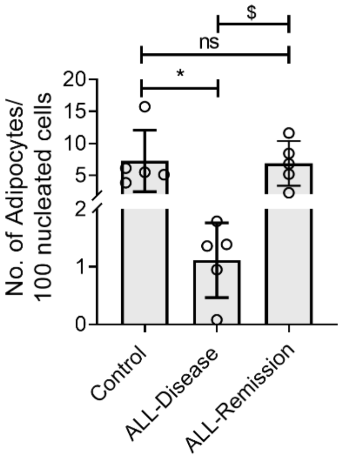


**Supplementary Figure 1. Relationship of marrow cellularity with BM adipocyte counts.** **a.** Scatter plot showing the correlation between the number of adipocytes and the total number of nucleated cells in healthy control (white circles) vs ALL disease BM biopsies (black squares). Statistical comparisons were performed using two-paired Pearson correlation. **b** Adipocyte numbers per 100 nucleated cells were quantified by Visiopharm analysis in healthy controls (n=5) and paired ALL disease vs ALL remission biopsies (n=5). Bars represent the mean ± SEM. *p= 0.04 and $^{\$}$p= 0.03 by one-way ANOVA with Tukey multiple comparison testing.

**a**

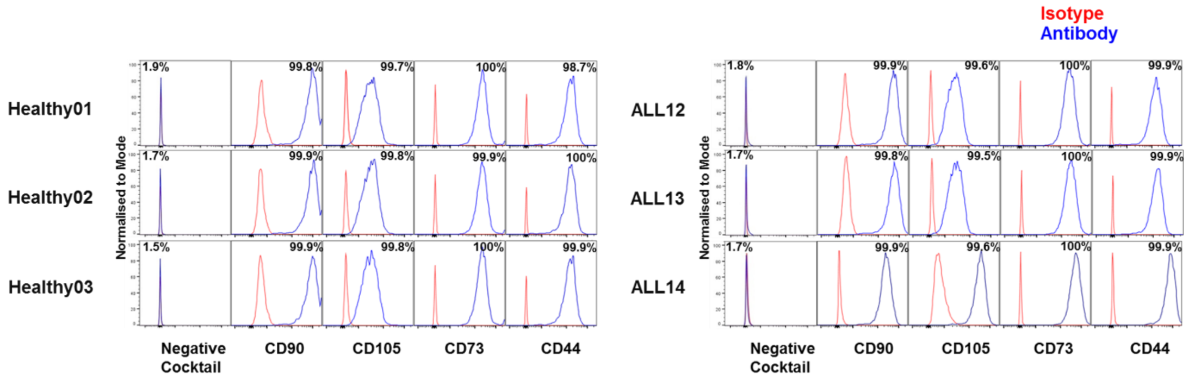


**Supplementary Figure 2. Immunophenotypic characterization of primary human BM-MSCs. a** Immunophenotype analysis of P1 plastic-adherent, fibroblast-like cells from representative healthy (n=3) and ALL-BMs (n=3) subjected to MSC culture conditions. The histograms show a panel of cell surface markers used to confirm MSC identity (1), i.e.,: positive staining for CD90, CD105, CD73 and CD44 associated with negative staining for endothelial or haematopoietic cell markers (CD45/CD34/Cd11b/CD19/HLA-DR). Estimates of marker positivity are indicated within each histogram.

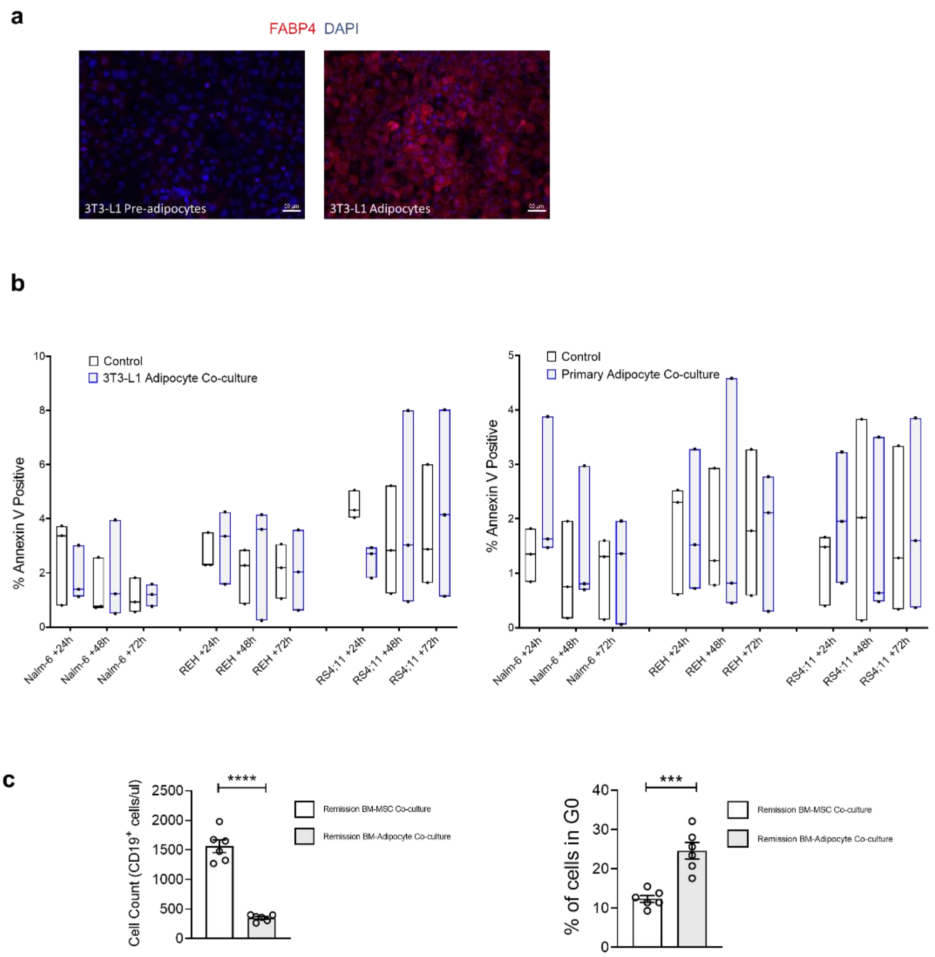


**Supplementary Figure 3. Phenotypic effects of *in vitro* adipocytes on ALL cells. a** Representative immunofluorescence images from two experiments showing adipocyte-specific FABP4 staining of 3T3-L1 adipocytes +10 days following *in vitro* differentiation. **b** Apoptosis induction in ALL cell lines subjected to monoculture vs coculture with 3T3-L1 adipocytes (left) or healthy primary BM-MSC-derived adipocytes (right) over time, as assessed by Annexin V and DAPI costaining in CD19+-gated populations. Data are represented as box plots where the middle line is the median and the lower and upper hinges correspond to the minimum and maximum mean plus SEM from three independent experiments, with duplicate testing performed for each condition (from **Fig. 3b**). Comparisons were performed between

control monoculture and coculture conditions at each respective time point by two-sided unpaired *t*-tests. None reached statistical significance ($p<0.05$). **c.** *In vitro* cell proliferation capacity (left) and dormant cell frequencies (right) of Nalm-6 cells after +72 hrs of coculture with primary BM-MSCs obtained at remission induction (remission BM-MSCs) vs remission BM-adipocytes differentiated from the respective remission BM-MSCs (from 2 independent donors). Graphs report the means ± SEM values of triplicate samples/per condition. Significance was assessed by two-sided unpaired *t*-tests. ****p= <0.0001 and ***p= 0.003

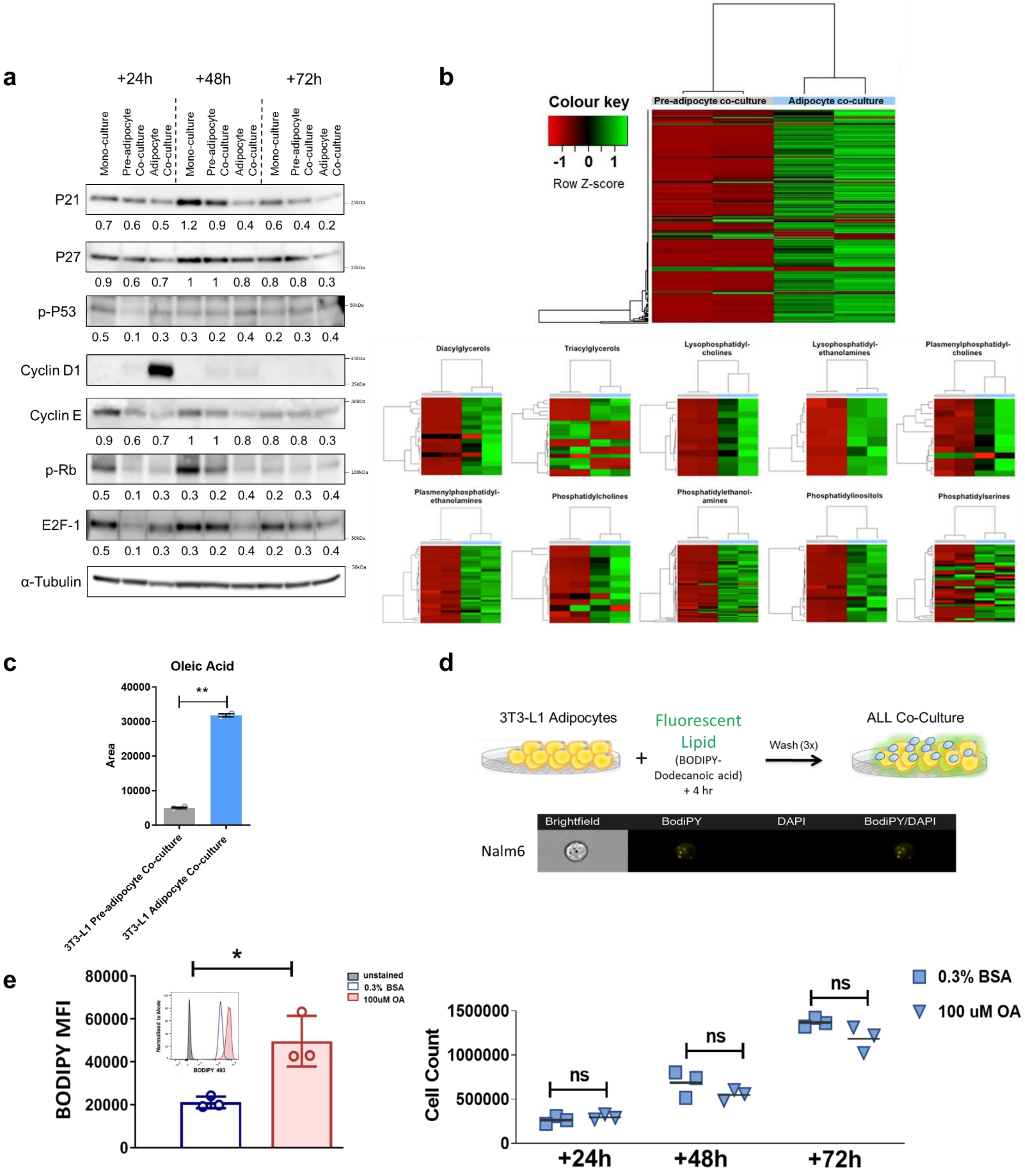

a
+24h
+48h
+72h
Mono-culture
Pre-adipocyte Co-culture
Adipocyte Co-culture
P21
0.7 0.6 0.5 1.2 0.9 0.4 0.6 0.4 0.2
P27
0.9 0.6 0.7 1 1 0.8 0.8 0.8 0.3
p-P53
0.5 0.1 0.3 0.3 0.2 0.4 0.2 0.3 0.4
Cyclin D1
Cyclin E
0.9 0.6 0.7 1 1 0.8 0.8 0.8 0.3
p-Rb
0.5 0.1 0.3 0.3 0.2 0.4 0.2 0.3 0.4
E2F-1
0.5 0.1 0.3 0.3 0.2 0.4 0.2 0.3 0.4
α-Tubulin
b
Colour key
-1 0 1
Row Z-score
Pre-adipocyte co-culture
Adipocyte co-culture
Diacylglycerols
Triacylglycerols
Lysophosphatidyl-cholines
Lysophosphatidyl-ethanolamines
Plasmenylphosphatidyl-cholines
Plasmenylphosphatidyl-ethanolamines
Phosphatidylcholines
Phosphatidylethanol-amines
Phosphatidylinositols
Phosphatidylserines
c
Oleic Acid
**
Area
40000
30000
20000
10000
0
3T3-L1 Pre-adipocyte Co-culture
3T3-L1 Adipocyte Co-culture
d
3T3-L1 Adipocytes
+
Fluorescent Lipid
(BODIPY-Dodecanoic acid)
+ 4 hr
Wash (3x)
ALL Co-Culture
Brightfield
BodiPY
DAPI
BodiPY/DAPI
Nalm6
e
*
BODIPY MFI
80000
60000
40000
20000
0
unstained
0.3% BSA
100uM OA
Normalised to Mode
BODIPY 493
Cell Count
1500000
1000000
500000
0
ns
ns
ns
+24h
+48h
+72h
0.3% BSA
100 uM OA

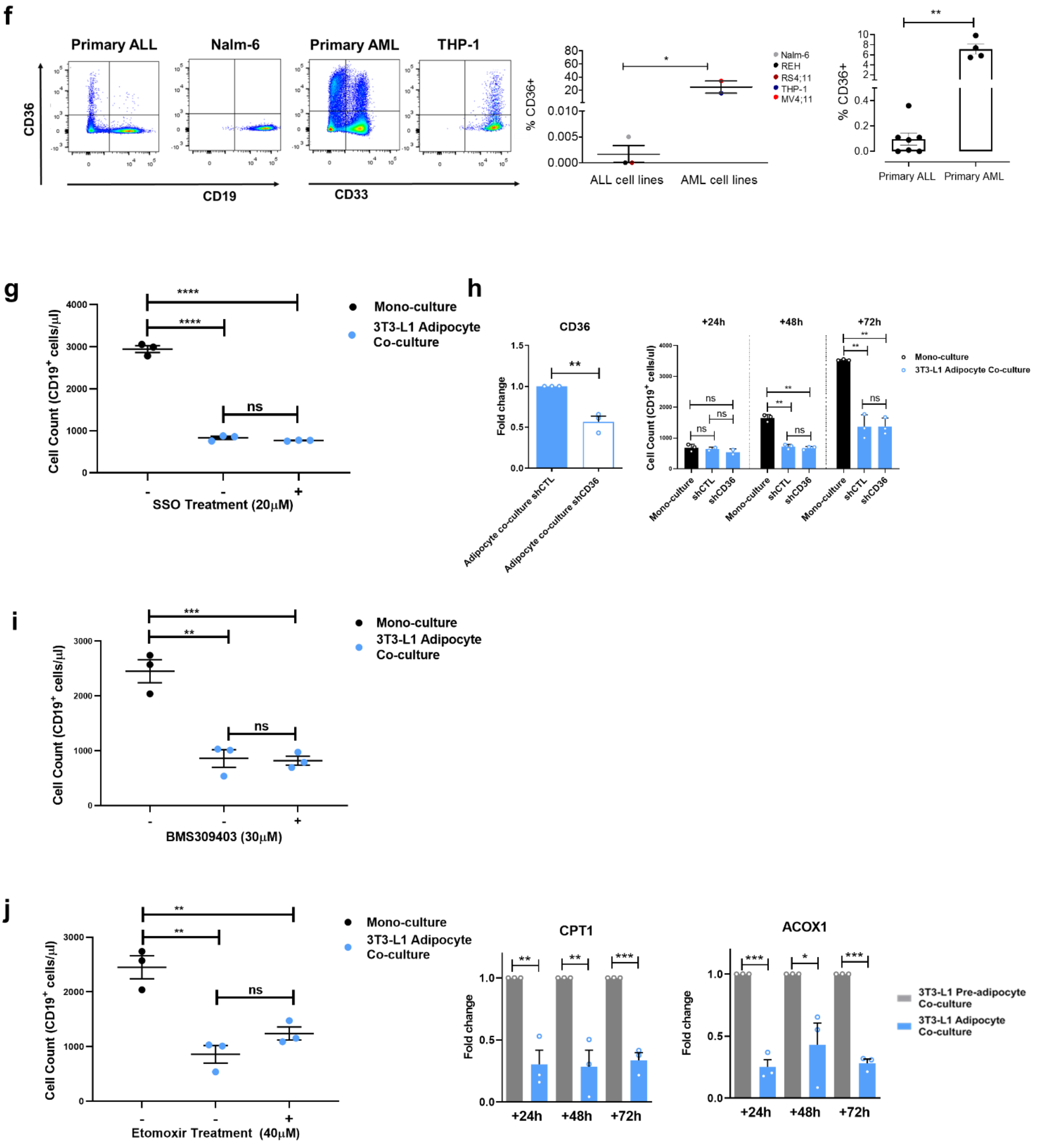
f
Primary ALL
Nalm-6
Primary AML
THP-1
CD36
CD19
CD33
% CD36+
ALL cell lines
AML cell lines
Nalm-6
REH
RS4;11
THP-1
MV4;11
Primary ALL
Primary AML
g
Cell Count (CD19+ cells/μl)
SSO Treatment (20μM)
Mono-culture
3T3-L1 Adipocyte Co-culture
h
CD36
Fold change
Adipocyte co-culture shCTL
Adipocyte co-culture shCD36
+24h
+48h
+72h
Cell Count (CD19+ cells/ul)
Mono-culture
shCTL
shCD36
Mono-culture
3T3-L1 Adipocyte Co-culture
i
Cell Count (CD19+ cells/μl)
BMS309403 (30μM)
Mono-culture
3T3-L1 Adipocyte Co-culture
j
Cell Count (CD19+ cells/μl)
Etomoxir Treatment (40μM)
Mono-culture
3T3-L1 Adipocyte Co-culture
CPT1
ACOX1
Fold change
+24h
+48h
+72h
3T3-L1 Pre-adipocyte Co-culture
3T3-L1 Adipocyte Co-culture

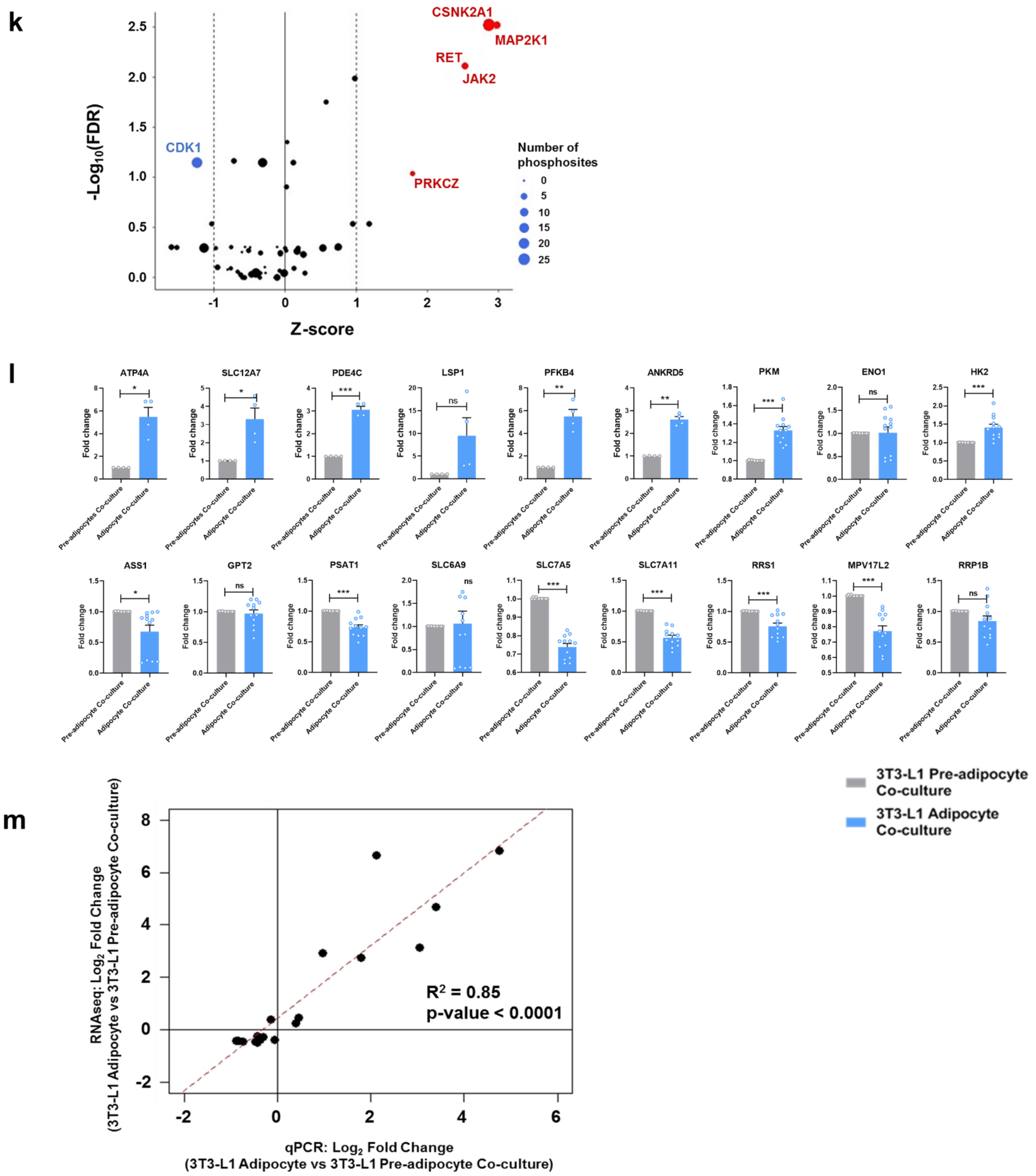

k
CSNK2A1
MAP2K1
RET
JAK2
CDK1
PRKCZ
Number of phosphosites
0
5
10
15
20
25
-Log10(FDR)
Z-score
l
ATP4A
SLC12A7
PDE4C
LSP1
PFKB4
ANKRD5
PKM
ENO1
HK2
ASS1
GPT2
PSAT1
SLC6A9
SLC7A5
SLC7A11
RRS1
MPV17L2
RRP1B
Fold change
Pre-adipocyte Co-culture
Adipocyte Co-culture
3T3-L1 Pre-adipocyte Co-culture
3T3-L1 Adipocyte Co-culture
m
R2 = 0.85
p-value < 0.0001
RNAseq: Log2 Fold Change
(3T3-L1 Adipocyte vs 3T3-L1 Pre-adipocyte Co-culture)
qPCR: Log2 Fold Change
(3T3-L1 Adipocyte vs 3T3-L1 Pre-adipocyte Co-culture)

n

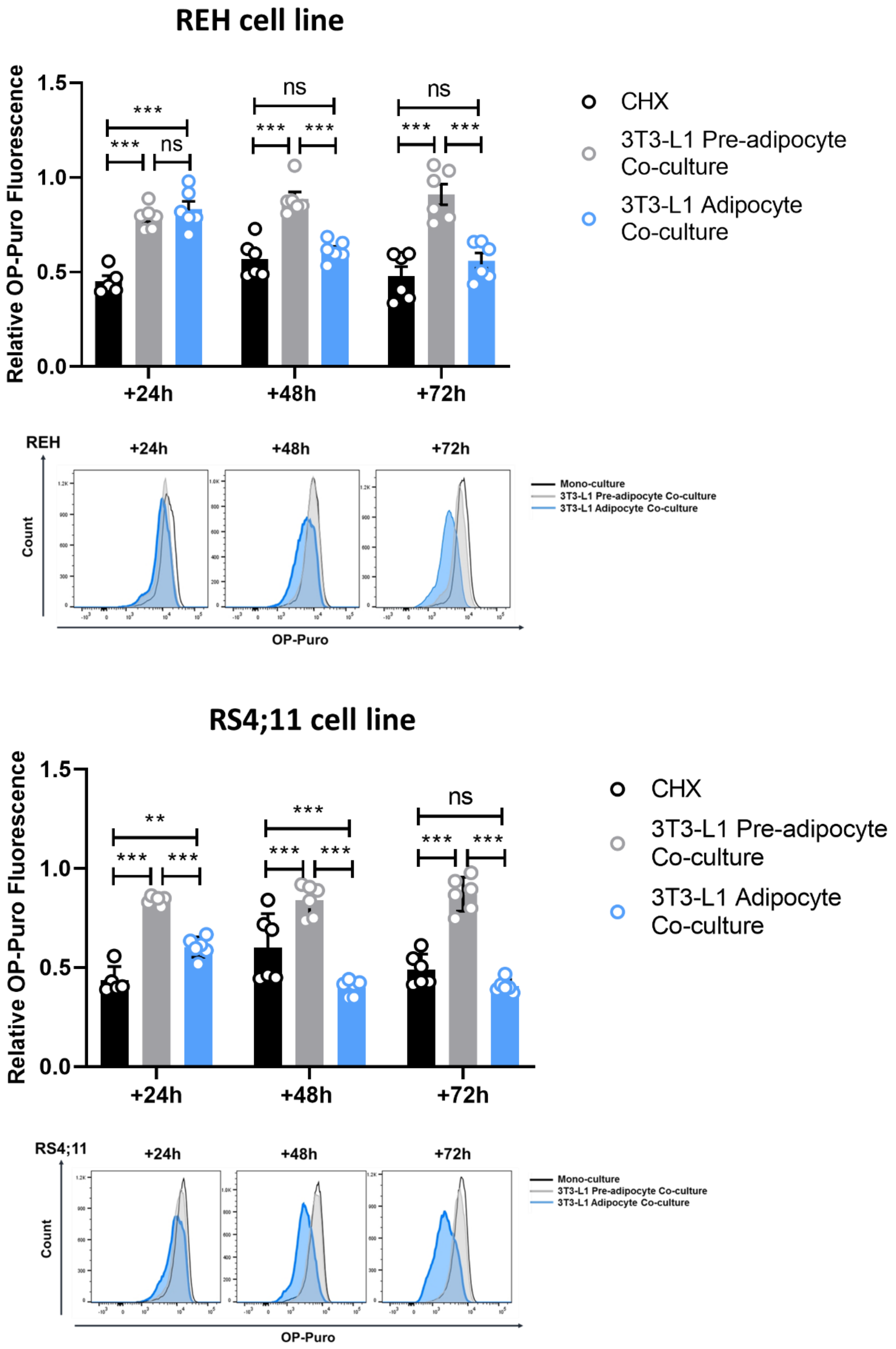
REH cell line
Relative OP-Puro Fluorescence
1.5
1.0
0.5
0.0
***
***
ns
ns
***
***
ns
***
***
+24h
+48h
+72h
CHX
3T3-L1 Pre-adipocyte Co-culture
3T3-L1 Adipocyte Co-culture
REH
Count
OP-Puro
Mono-culture
3T3-L1 Pre-adipocyte Co-culture
3T3-L1 Adipocyte Co-culture
RS4;11 cell line
Relative OP-Puro Fluorescence
1.5
1.0
0.5
0.0
**
***
***
***
***
***
ns
***
***
+24h
+48h
+72h
CHX
3T3-L1 Pre-adipocyte Co-culture
3T3-L1 Adipocyte Co-culture
RS4;11
Count
OP-Puro
Mono-culture
3T3-L1 Pre-adipocyte Co-culture
3T3-L1 Adipocyte Co-culture

o

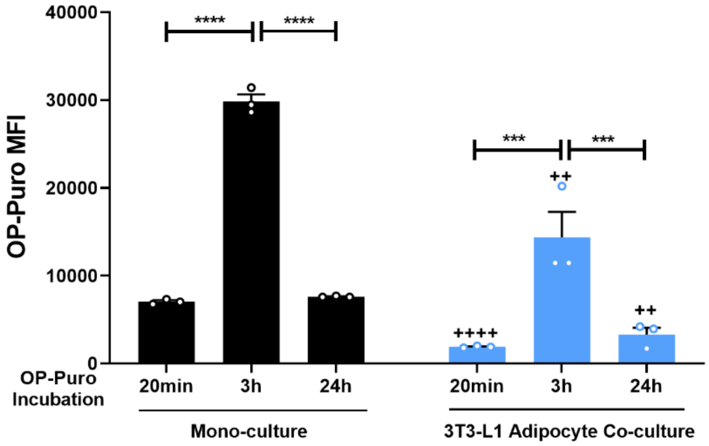
40000
30000
20000
10000
0
OP-Puro MFI
****
****
***
***
++
++
++++
OP-Puro Incubation
20min
3h
24h
20min
3h
24h
Mono-culture
3T3-L1 Adipocyte Co-culture

p

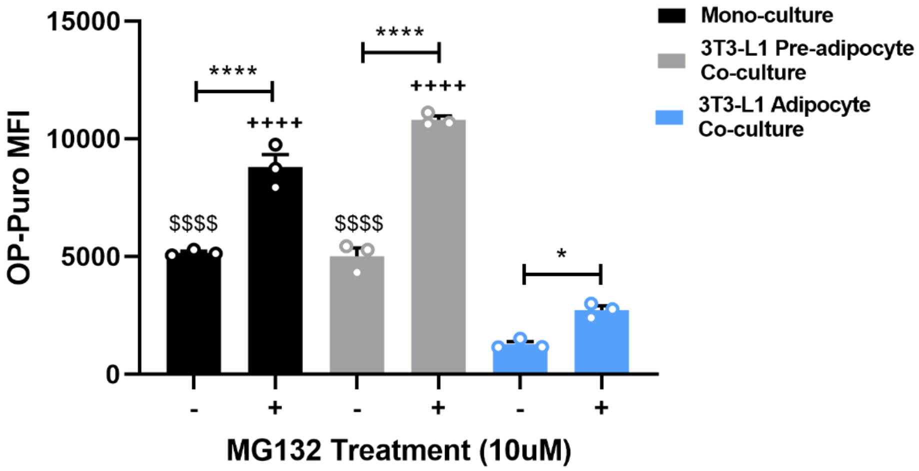
15000
10000
5000
0
OP-Puro MFI
****
****
++++
++++
$$$$
$$$$
*
-
+
-
+
-
+
MG132 Treatment (10uM)
Mono-culture
3T3-L1 Pre-adipocyte Co-culture
3T3-L1 Adipocyte Co-culture

q

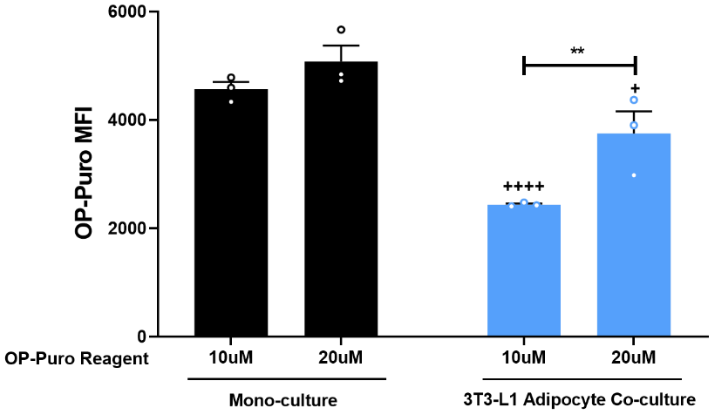
6000
4000
2000
0
OP-Puro MFI
**
+
++++
OP-Puro Reagent
10uM
20uM
10uM
20uM
Mono-culture
3T3-L1 Adipocyte Co-culture

**r**

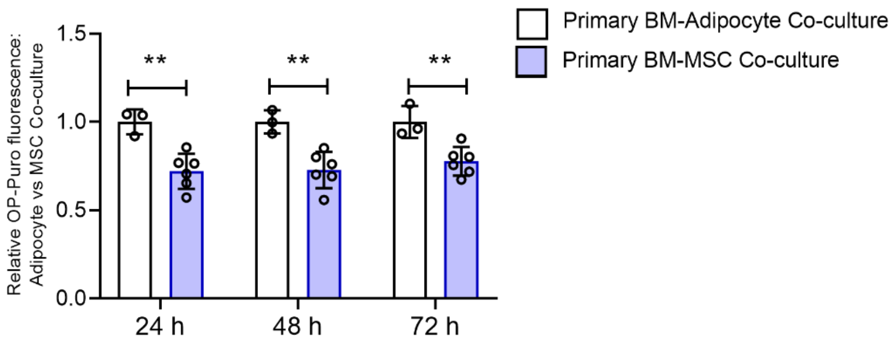

Primary BM-Adipocyte Co-culture
Primary BM-MSC Co-culture
Relative OP-Puro fluorescence: Adipocyte vs MSC Co-culture
1.5
1.0
0.5
0.0
**
**
**
24 h
48 h
72 h


**s**

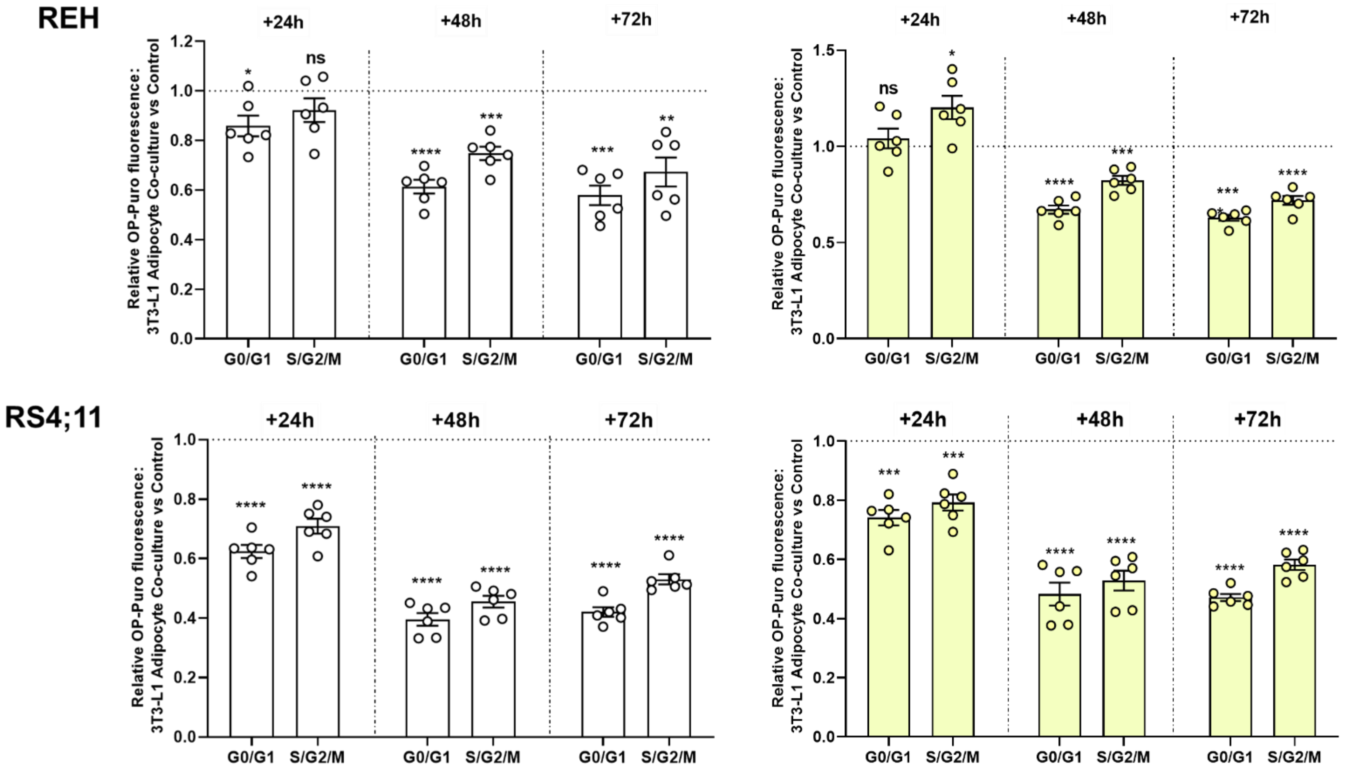

REH
RS4;11
Relative OP-Puro fluorescence: 3T3-L1 Adipocyte Co-culture vs Control
+24h
+48h
+72h
G0/G1
S/G2/M
*
ns
****
***
**

**t**

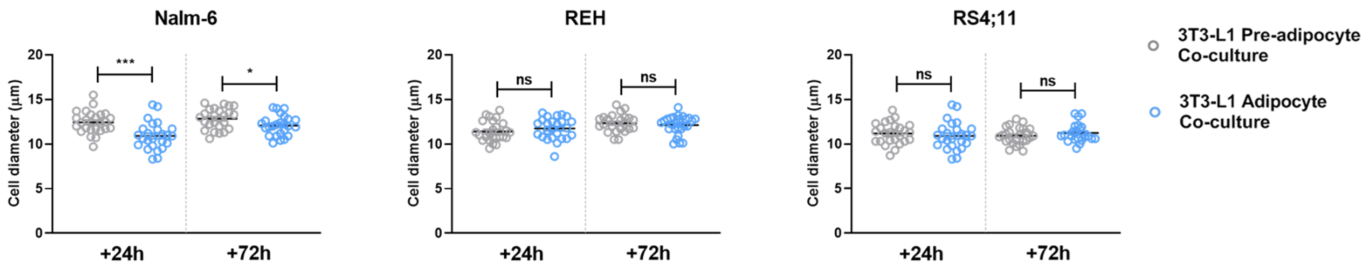

Nalm-6
REH
RS4;11
Cell diameter (µm)
0
5
10
15
20
***
*
ns
+24h
+72h
3T3-L1 Pre-adipocyte Co-culture
3T3-L1 Adipocyte Co-culture


**u**

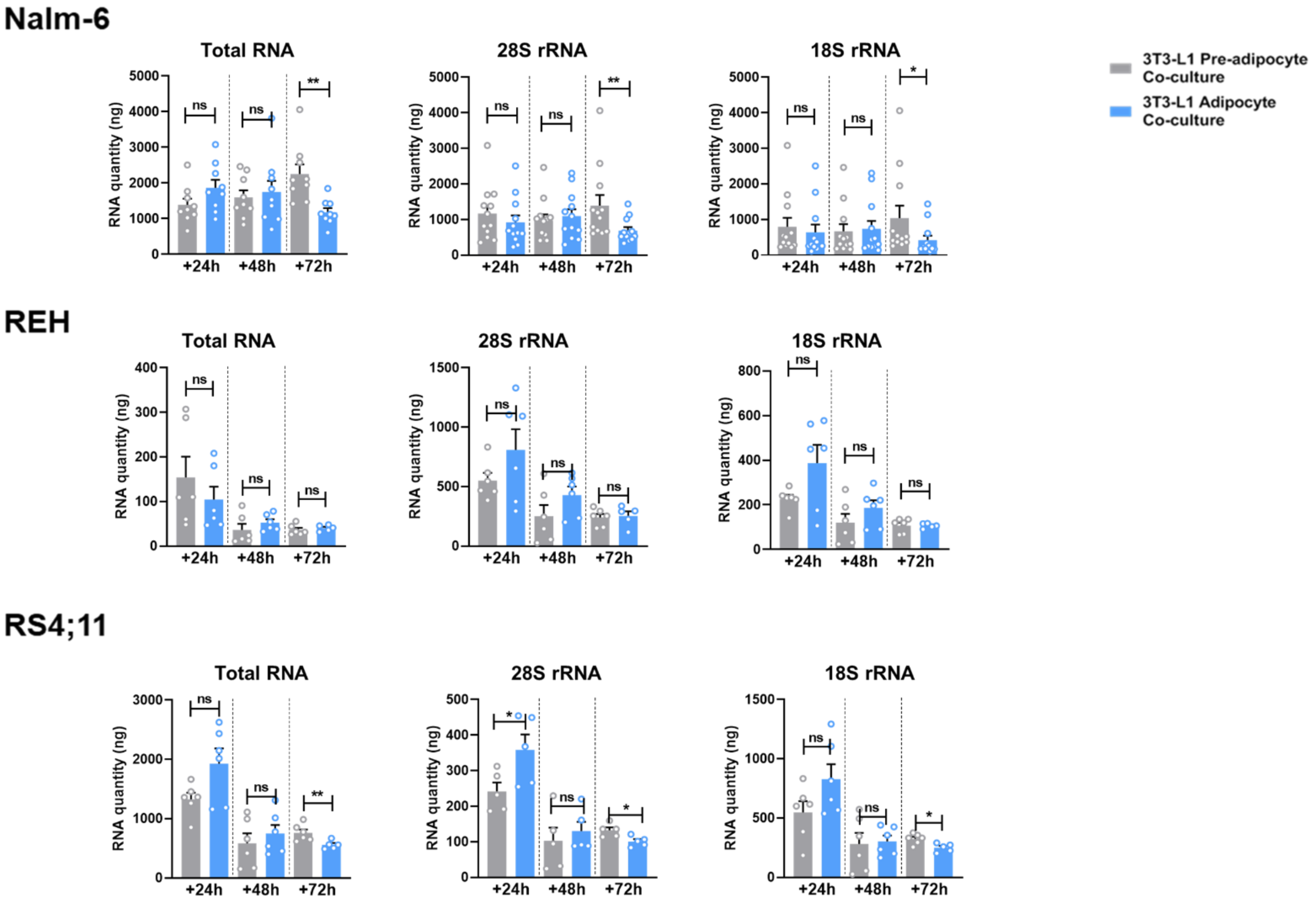

Nalm-6
Total RNA
28S rRNA
18S rRNA
RNA quantity (ng)
0
1000
2000
3000
4000
5000
ns
**
*
+24h
+48h
+72h
3T3-L1 Pre-adipocyte Co-culture
3T3-L1 Adipocyte Co-culture
REH
Total RNA
28S rRNA
18S rRNA
0
100
200
300
400
500
1000
1500
600
800
RS4;11
Total RNA
28S rRNA
18S rRNA
3000

v

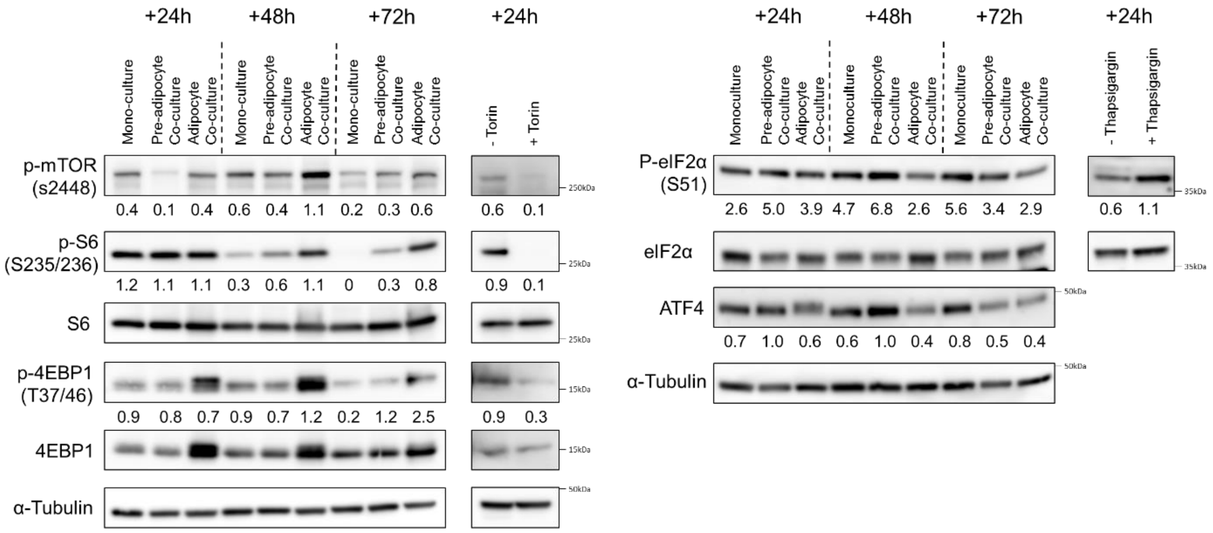

+24h
+48h
+72h
+24h
Mono-culture
Pre-adipocyte Co-culture
Adipocyte Co-culture
- Torin
+ Torin
p-mTOR (s2448)
0.4 0.1 0.4 0.6 0.4 1.1 0.2 0.3 0.6
0.6 0.1
p-S6 (S235/236)
1.2 1.1 1.1 0.3 0.6 1.1 0 0.3 0.8
0.9 0.1
S6
p-4EBP1 (T37/46)
0.9 0.8 0.7 0.9 0.7 1.2 0.2 1.2 2.5
0.9 0.3
4EBP1
α-Tubulin
Monoculture
- Thapsigargin
+ Thapsigargin
P-eIF2α (S51)
2.6 5.0 3.9 4.7 6.8 2.6 5.6 3.4 2.9
0.6 1.1
eIF2α
ATF4
0.7 1.0 0.6 0.6 1.0 0.4 0.8 0.5 0.4
α-Tubulin


w

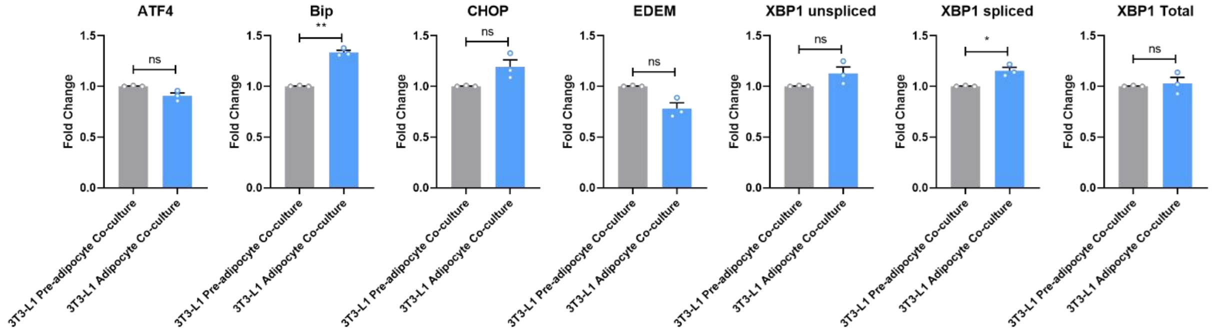

ATF4
Bip
CHOP
EDEM
XBP1 unspliced
XBP1 spliced
XBP1 Total
Fold Change
ns
**
*
3T3-L1 Pre-adipocyte Co-culture
3T3-L1 Adipocyte Co-culture


x

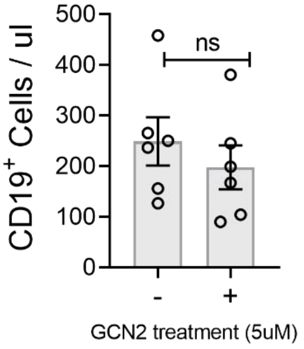

CD19+ Cells / ul
ns
-
+
GCN2 treatment (5uM)

**Supplementary Figure 4. Role of cell cycle checkpoint and lipid flux in adipocyte suppression of ALL. a** Western blot showing cell cycle arrest proteins (P21, P27 and p-P53) and cell cycle regulators (Cyclin D1, Cyclin E, p-Rb and E2F-1). Lysates were prepared from equal cell numbers ($5x10^6$ cells). Expression was calculated as the ratio of the density of the target protein to that of α-Tubulin. Blots are representative of two independent experiments showing similar results. **b** Heatmap of the global lipidome in Nalm-6 cells subjected to 3T3-L1 adipocyte vs 3T3-L1 preadipocyte coculture at +24 h (top). Individual heat maps (below) represent data for each of the major lipid classes. *Z*-score, red: ≤-1, green: ≥+1). **c** Intracellular oleic acid abundance in Nalm-6 cells subjected to 3T3-L1 adipocyte (blue) vs 3T3-L1 preadipocyte (grey) coculture at +24 h. The results are a subset analysis of the global lipidomics analysis described in **b** **p=0.0017. **d** Representative image stream analysis showing translocation of BODIPY-dodecanoic acid (BODIPY), a fluorescent fatty acid analogue, from labelled 3T3-L1 adipocytes to Nalm-6 cells after +24 h coculture. 3T3-L1 adipocytes were pulsed with BODIPY for 4 h, washed and then incubated with Nalm-6. **e.** Intracellular lipid content of Nalm-6 cells following supplementation with 100 µM oleic acid (OA) as quantified by BODIPY 493 fluorescence (left). The inset shows the corresponding flow cytometric histograms. Corresponding growth data of Nalm-6 cells under OA supplementation (right). *p=0.0466. **f** Representative flow cytometric analysis of the fatty acid transporter CD36 in ALL cell lines (Nalm-6, REH and RS4;11, white), AML cell lines (MV4;11 and THP-1, black), primary ALL samples (n=7, white) and primary AML samples (n=4, grey). Each biologically independent sampled assayed once. *p= 0.04, **p=0.006. **g** Growth outcomes of Nalm-6 cells at +72 h following coculture with 3T3-L1 adipocytes in the presence (blue) or absence (black) of sulfo-N-succinimidyl oleate (a CD36 inhibitor). **h** Knockdown efficiency and specificity in Nalm-6 cells transduced with CD36 shRNA (shCD36) or control shRNA (shCTL) assessed by RT-qPCR after 72 hrs of coculture with 3T3-L1 adipocytes (left) and corresponding growth data (right). Data are triplicates from one experiment. Statistical comparisons performed using two-sided unpaired *t*-test for RT-qPCR. **p<0.0033. Two-way ANOVA followed by Tukey's test for multiple comparisons for growth

data. **p<0.05. **i.** Growth outcomes of Nalm-6 cells at +72 h following coculture with 3T3-L1 adipocytes in the presence (blue) or absence (black) of 30 µM BMS309403 (FABP4 inhibitor). Monoculture vs 3T3-L1 adipocyte coculture: **p=0.0011; monoculture vs 3T3-L1 adipocyte coculture + BMS treatment: ***p=0.0009. **j** Growth outcomes of Nalm-6 cells at +72 h following coculture with 3T3-L1 adipocytes in the presence (blue) or absence (black) of 40 µM etomoxir from one experiment tested in triplicate (left) and RT-qPCR analysis of fatty acid oxidation genes, CPT1 and ACOX1, in Nalm-6 cells cocultured with either 3T3-L1 preadipocytes (grey) or 3T3-L1 adipocytes (blue) over time tested in triplicates from three independent experiments (right). Statistical comparisons were performed using two-sided *t*-tests and one-way ANOVA followed by Tukey's test for multiple comparisons respectively. **k** Volcano plot comparing  kinase-substrate enrichment analysis (KSEA) results in Nalm-6 cells cocultured with 3T3-L1 adipocytes vs 3T3-L1 preadipocytes at +24 h. Kinase substrates with a *Z*-score≤-1 and FDR q-value<0.05 are represented in blue. Kinase substrates with a *Z*-score≥+1 and FDR q-value<0.05 are represented in red. **l** RT-qPCR validation of significantly differentially regulated genes (p-value<0.005 and $\log_2$ FC≥+0.3 or $\log_2$ FC≤-0.3) identified by RNAseq in Nalm-6 cells cocultured with 3T3-L1 adipocytes (blue) vs 3T3-L1 preadipocytes (grey) at +24 h. Data are inclusive of the same RNA-seq sample. RT-qPCR validated 13/18 significant genes from three independent experiments. **m** Scatter plot showing the correlation between significant genes identified by RNAseq (p-value<0.005 and $\log_2$ FC≥+0.3 or $\log_2$ FC≤-0.3) in Nalm-6 cells cocultured with 3T3-L1 adipocytes vs 3T3-L1 preadipocytes at +24 h with RT-qPCR analysis. **n** Quantification of nascent protein synthesis in REH (top) and RS4;11 (bottom) cells cocultured with 3T3-L1 adipocytes over time normalised to monoculture. **o**  OP-Puro fluorescence at 20 min, 3 or 24 h after OP-Puro administration in Nalm-6 cells at +48 h under various culture conditions. Comparisons of monoculture (black) vs 3T3-L1 adipocyte coculture (blue) were performed using  two-sided unpaired *t* test (++p<0.01, ++++p<0.0001). Comparisons within cell culture conditions were assessed using two-way ANOVA followed by Tukey's test for multiple comparisons (***p<0.001, ****p<0.0001). **p** OP-Puro fluorescence in Nalm-6 cells under the indicated cell

culture conditions at +72 h in the presence of MG132 to inhibit proteasome activity. Significance is indicated by + for differences between adipocyte coculture and other cell culture conditions under MG132 drug treatment ++++ $p<0.0001$, $ for differences between adipocyte coculture and other cell culture conditions without drug treatment ($$$$ $p<0.0001$) and * for differences within each cell culture condition (*$p<0.05$, ****$p<0.0001$). **q** OP-Puro fluorescence in Nalm-6 cells under the indicated cell culture conditions at +24 h using different concentrations of OP-Puro. Significance is indicated by + for differences between monoculture (black) and adipocyte coculture (blue) (+$p<0.05$, ++$p<0.01$) and by * for differences within the same cell culture condition (*$p<0.05$). **r** Quantification of nascent protein synthesis in Nalm-6 cells cocultured with primary BM- adipocytes over time normalised to coculture with primary BM-MSC (two independent healthy donors). **s** OP-Puro incorporation in vitro in REH (top) and RS4;11 (bottom) in G0/G1 vs S/G2/M normalised to monoculture (unfilled symbols-left) or 3T3-L1 preadipocytes (yellow symbols-right). **t** Cell diameters (µm) in the indicated ALL cell lines following coculture with either 3T3-L1 preadipocytes (grey; n= 23-25 cells) or 3T3-L1 adipocytes (blue; n=23-25). DAPI nuclear staining and fluorescence microscopy using a Nikon Ci camera with ImageJ (version 1.50b, USA) software. Nalm-6 at +24 h p=0.0004, Nalm-6 at +72 h p=0.026). **u** Total RNA, 28S rRNA and 18S rRNA content in 500,000 Nalm-6 (n=9), REH (n=6) or RS4;11 (n=6) cells following coculture with either 3T3-L1 preadipocytes (grey) or 3T3-L1 adipocytes (blue) over time. **v** Western blot analysis of p-mTOR, p-S6 substrate, total S6 substrate, p-4EBP1 and total 4EBP1 antibodies (left) or p-eIF2α, total eIF2α and ATF4 antibodies (right) in Nalm-6 cells ($5x10^6$ cells) in monoculture or following coculture with either 3T3-L1 preadipocytes or 3T3-L1 adipocytes over time. Nalm-6 cells treated with Torin (1 µM) or thapsigargin (250 nM) serve as positive controls. Protein expression ratios were calculated by comparing the phosphorylated forms with the total forms where applicable or with α-tubulin. Blots are representative of two independent experiments showing similar results. **w** RT-qPCR analysis of ATF4, Bip, CHOP, EDEM, XBP1 unspliced, spliced and total ER stress-related genes in Nalm-6 cells cocultured with either 3T3-L1 preadipocytes (grey) or 3T3-L1 adipocytes (blue)

for +24 h.  **x** Growth outcomes in GCN2ib (5 µg/mL) vs vehicle  treated Nalm-6 cells +72 h in 3T3-L1 adipocyte-culture. All data are means and SEM values of independent experiments (n=1 in **b,c** assessing duplicates**, e, g, h, i, l, o, p, q, t, w** assessing triplicates, n = 2 in **n, r, s, x** assessing triplicates,  n = 3 in **l** assessing triplicates, and in **u** assessing 2-3 replicates. Statistical significance was assessed using 2-sided unpaired *t* tests (**c, e g, h, l, n, s, t, u, w, x),** Mann-Whitney *U*-tests (**f**)**,** one-way ANOVA followed by Tukey's test for multiple comparisons (**g, h, i, l, p, q, r**) 2-sided Pearson correlation (**m**)**.** For **h, j**, **o**, statistical analysis is described under the relevant section. *p<0.05, **p<0.01, ***p<0.001****p<0.0001 or precise values are given. ns, not significant.

**Supplementary discussion for fig 4b-j**

To investigate the involvement of lipid influx mechanisms in the adipocyte-mediated suppression of ALL, we first assessed lipid response in ALL cells under 3T3-L1 adipocyte coculture using global lipidomic analysis at +24 h. We observed a broad increase in the overall lipid content of ALL cells at this time **(Supplementary Fig 4b),** which involved all major lipid classes, including oleic acid, a major class of free fatty acids **(Supplementary Fig 4c)**. This increase in the cellular pool of fatty acids reflected, in part, uptake of exogenous fatty acids directly from the 3T3-L1 adipocyte stroma **(Supplementary Fig 4d),** consistent with a well-known metabolic crosstalk (2, 3). To functionally evaluate the effects of fatty acid influx on ALL, we performed complementary assessments. First, we conditioned Nalm-6 ALL cells with exogenous oleic acid, which significantly increased the intracellular lipid content but did not phenocopy the adipocyte growth-suppressive response **(Supplementary Fig 4e)**. Second, we targeted fatty acid transport mechanisms as a broader approach to assaying the role of fatty acid transfer, focusing initially on CD36 receptor-dependent fatty acid transport given its functional link with AML stem cell quiescence(2). We determined that ALL tumours had very low levels of CD36 receptor expression both in ALL cell lines as well as primary tumours **(Supplementary Fig 4f)** whilst confirming that in AML it was variably positive as previously described (2, 4)**.** Together with the finding that knockdown of CD36 either pharmacologically **(Supplementary Fig 4g)** or genetically **(Supplementary Fig 4h)** failed to abrogate the adipocyte suppression of ALL confirmed a non-prominent role of this pathway in the 3T3-L1 adipocyte effect. Similarly, pharmacological blockade of FABP4, an alternative mechanism of fatty acid transport, also failed to rescue the suppressive effect of adipocytes on ALL growth **(Supplementary Fig 4i)**. Third, as an additional validation, the CPT1 inhibitor, etomoxir was applied to block fatty acid oxidation (FAO), and therefore fatty acid metabolism which similarly did not lead to rescue of the adipocyte suppressive effect **(Supplementary Fig 4j).** Together, these data indicate that fatty acid flux is unlikely to be the main mediator of the effect of adipocytes on ALL suppression.

**a**

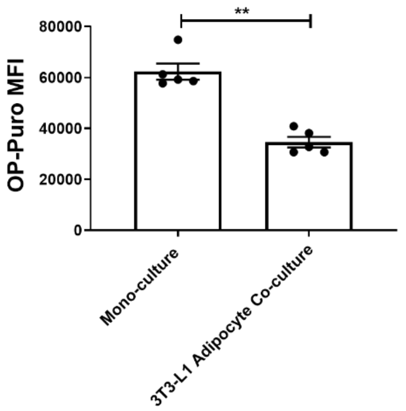

**
80000
60000
40000
20000
0
OP-Puro MFI
Mono-culture
3T3-L1 Adipocyte Co-culture


**b**

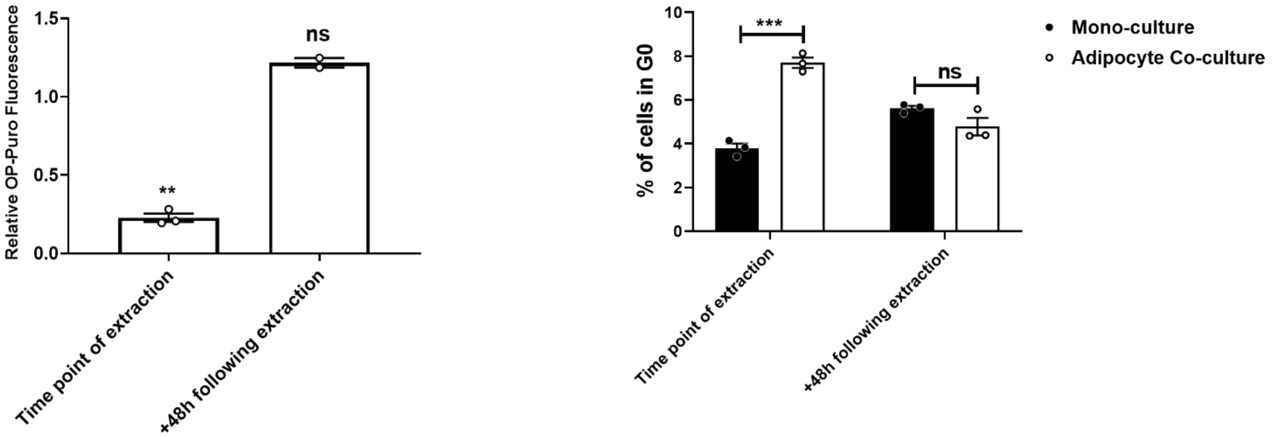

1.5
1.0
0.5
0.0
Relative OP-Puro Fluorescence
**
ns
Time point of extraction
+48h following extraction
10
8
6
4
2
0
% of cells in G0
***
ns
Time point of extraction
+48h following extraction
Mono-culture
Adipocyte Co-culture


**c**

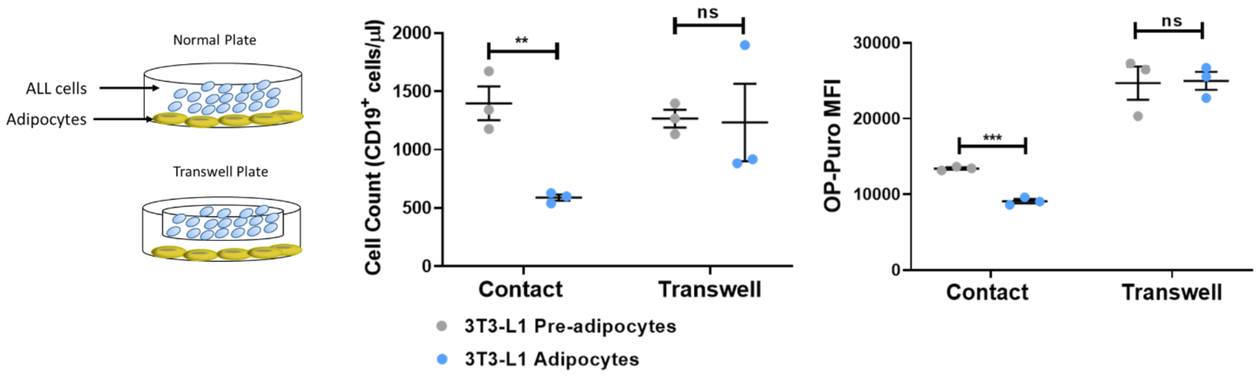

Normal Plate
ALL cells
Adipocytes
Transwell Plate
2000
1500
1000
500
0
Cell Count (CD19+ cells/μl)
**
ns
Contact
Transwell
3T3-L1 Pre-adipocytes
3T3-L1 Adipocytes
30000
20000
10000
0
OP-Puro MFI
***
ns
Contact
Transwell

d

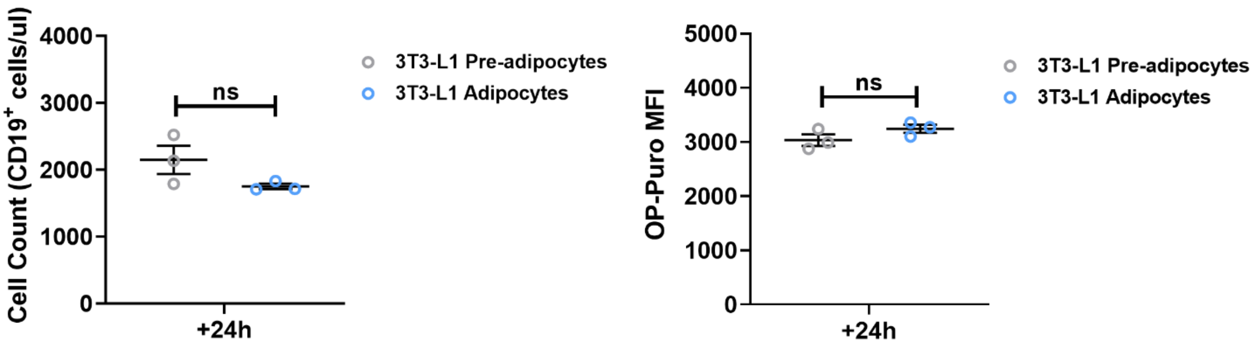


e

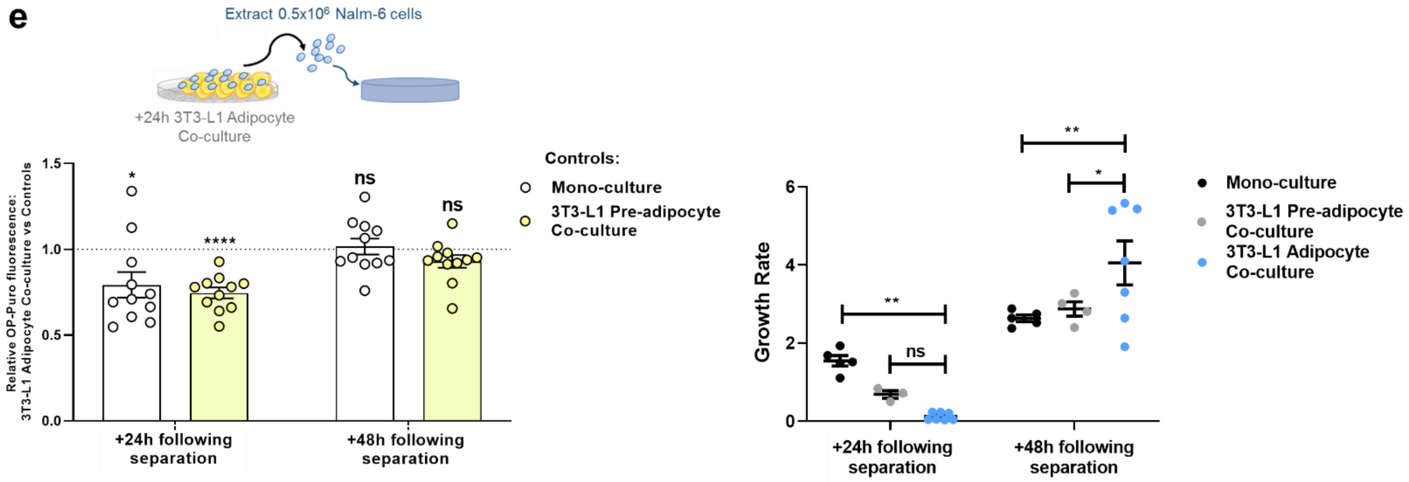


**Supplementary Figure 5. Requirement for cell contact in adipocyte-induced ALL suppressive outcomes. a** OP-Puro fluorescence in untreated Nalm-6 cells at +4 h following extraction from monoculture or 3T3-L1 adipocytes showing persistent repression of protein translation at this point. **p=0.0079. **b** OP-Puro fluorescence (left) and G0 frequency (right) in Nalm-6 cells after 48 h recovery from 3T3-L1 adipocyte coculture and prior to experiments described in **Fig. 5b.** OP-Puro fluorescence is expressed relative to monoculture. **p=0.0018, ***p=0.0003. **c** Growth outcome of Nalm-6 cells in the presence of 3T3-L1 adipocytes (blue) or 3T3-L1 preadipocytes (grey) using a Transwell coculture system (left). Corresponding OP-Puro analysis (right). **p=0.0054, ***p=0.0002. **d** Growth outcomes (left) and OP-Puro fluorescence (right) of Nalm-6 cells at +24 h following coculture with 3T3-L1 adipocyte-conditioned medium (blue) or 3T3-L1 preadipocyte-conditioned medium (grey). **e** Recovery of nascent protein synthesis (left) and cellular growth (right) in Nalm-6 cells released from the

adipocyte microenvironment. Nalm-6 cells were cocultured with 3T3-L1 adipocytes for +24 h, and $0.5x10^6$ cells were extracted for reculture under ALL cell line conditions alone. Subsequently, OP-puro incorporation and cellular growth at +24 and +48 h following 3T3-L1 adipocyte extraction were assessed relative to Nalm-6 cells separated at +24 h from monoculture or preadipocyte coculture. Bars are the mean ± SEM of three independent experiments performed with 3-4 replicates (OP-puro incorporation) or two independent experiments performed with 2-3 replicates (cellular growth). Comparisons of OP-puro fluorescence were performed using two-sided unpaired *t*-tests. Cellular growth comparisons were performed using mixed-effects analysis followed by Dunnett's test for multiple comparisons. *p<0.05, **p<0.01, ****p<0.0001. All data are presented as mean values ± SEM values of independent experiments (n=2 in **a** with 2-3 replicates**,** n = 1 with triplicates in **b, c and d).** Statistical significance was assessed by two-sided Mann-Whitney *U*-test (**a**), 2-sided unpaired *t* tests (**b**,**c, d).** *p<0.05 **p<0.01, ***p<0.001, ****p<0.0001 or the precise p-value where indicated.

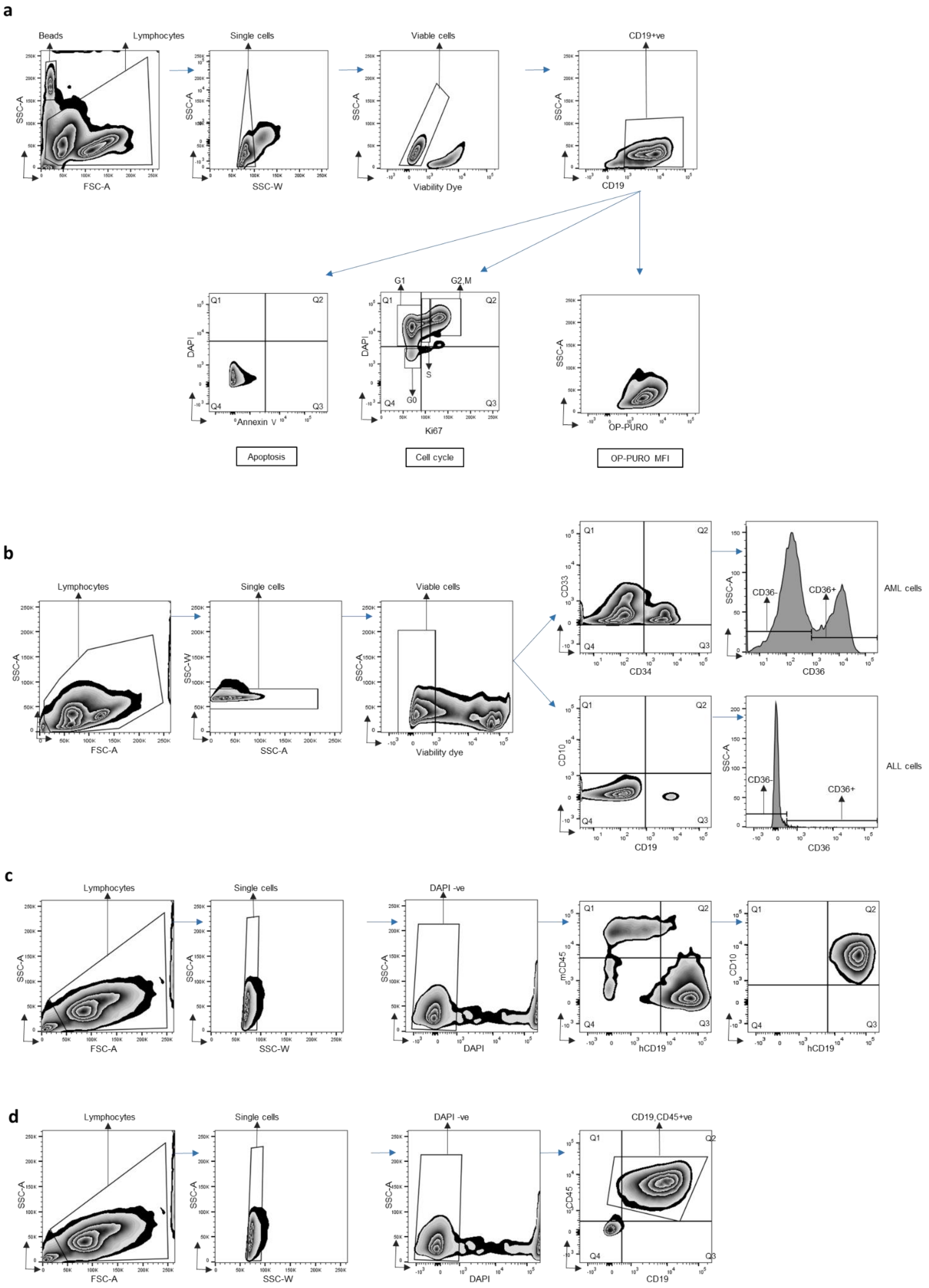
a
Beads
Lymphocytes
SSC-A
FSC-A
Single cells
SSC-W
Viable cells
Viability Dye
CD19+ve
CD19
Q1
Q2
Q3
Q4
DAPI
Annexin V
Apoptosis
G1
G2,M
S
G0
Ki67
Cell cycle
OP-PURO
OP-PURO MFI
b
Lymphocytes
Single cells
SSC-W
SSC-A
Viable cells
Viability dye
CD33
CD34
CD36-
CD36+
CD36
AML cells
CD10
CD19
ALL cells
c
Lymphocytes
Single cells
DAPI -ve
DAPI
mCD45
hCD19
CD10
d
Lymphocytes
Single cells
DAPI -ve
CD19,CD45+ve
CD45
CD19

**Supplementary Figure 6. Flow Cytometry Gating strategies. a.** Gating strategy for in vitro analysis of CD19+ cells, cell count, annexin positive cells, cell cycle or OP-Puro MFI presented in **Fig. 3b, 3c, 3h, 3i, 4e, 4f, 4g, 4i, 4j** and **Supplementary Fig. S3b, S3c, S4g, S4i, S4j, S4n-s, S4x, S5a-e. b** Gating strategy for CD36+ cells presented in Supplementary **Fig. S4j. c-d** Gating strategy for cell line (**c**) and primary ALL (**d**) xenograft assessments presented in **Fig. 3f and 3g**.

Supplementary Tables

| Adipocyte Size (µm$^2$) | | | |
|---|---|---|---|
| Patient | ALL-Diagnosis | ALL-Remission | p-value |
| ALL01 | 323.7 ± 18.87 | 1326 ± 28.53 | **** |
| ALL02 | 859.7 ± 55.32 | 992.3 ± 36.6 | ns |
| ALL03 | 159.8 ± 18.35 | 540.5 ± 58.72 | **** |
| ALL04 | 199.1 ± 4.837 | 272.4 ± 39.6 | ns |
| ALL05 | 272.4 ± 39.6 | 610.7 ± 47.56 | **** |
| ALL06 | 400.5 ± 11.86 | 1141 ± 22.98 | **** |
| ALL07 | 221.2 ± 19.75 | 1028 ± 513.9 | **** |
| ALL08 | 125.7 ± 6.444 | 724.9 ± 24.64 | **** |

**Supplementary Table 1. Adipocyte size in matched ALL diagnosis and ALL remission biopsies (n=8) quantified by Visiopharm analysis**. Data present the means and SEM value. Statistical comparisons were performed by two-sided Mann-Whitney *U* tests (****p<0.0001).

| | No. of samples tested | No. of samples with successful MSC isolation | Success rate |
|---|---|---|---|
| **Healthy** | 14 | 14 | 100% |
| **ALL** | 16 | 12 | 75% |

p=0.1046 (Healthy vs ALL)

**Supplementary Table 2. Frequency of MSC generation at P1 in consecutively tested BM samples**. Statistical analysis was performed using an unpaired two-sided *t* test.

| UPN | Age (yrs) | Sex | Disease status | Sample type | Cytogenetics | Major Cytogenetic group/risk status | Experiments |
|---|---|---|---|---|---|---|---|
| **ALL01** | 64 | M | Diagnosis | BM | [1]Near tetraploid clone | Other/Standard | AA |
| **ALL02** | 53 | M | Diagnosis | BM | 46,XY,del(9)(p13) | del(9p)/Standard | AA |
| **ALL03** | 58 | M | Diagnosis | BM | 55,XY,+X,+2,+4,+4,+6,der(9)t(9;22)(q34;q11.2),+10,+?20,+21, ider(22)(q10)t(9;22),+ider(22),inc | t(9;22)/High | AA |
| **ALL04** | 43 | M | Diagnosis | BM | 46,XY,t(9;22)(q34;q11.2),del(10)(q22q26) | t(9;22)/High | AA |
| **ALL05** | 25 | F | Diagnosis | BM | 46,XX,t(9;22)(q34;q11.2) | t(9;22)/High | AA |
| **ALL06** | 36 | F | Diagnosis | BM | 46,XX | Standard | AA |
| **ALL07** | 38 | F | Diagnosis | BM | 46,XX | Standard | AA |
| **ALL08** | 25 | M | Diagnosis | BM | 42,XY,-3,-7,del(9)(p13),-13,-15,-17,+mar | del(9p)/Standard | AA |
| **ALL09** | 29 | M | Post Treat | BM | 46,XY,t(9;22)(q34;q11.2) | t(9;22)/High | IHC |
| **ALL10** | 21 | M | Post Treat | BM | 44~46,XY,-4,der(?)t(5;?)(q11.2;?),add(9)(q34),der(11)t(11;13)(q23;q?21),-13,-17,-17,+3mar | Complex/High | IHC |
| **ALL11** | 20 | M | Post Treat | BM | 46,XY,del(6)(q1q2) | Other/Standard | IHC |
| **ALL12** | | | Diagnosis | BM | No information available | | IP, MSC, IF |
| **ALL13** | 25 | M | Diagnosis | BM | 47~49,XY,add(4)(q2),-6,add(9)(q2),-13,-15,add(16)(p11.2), +5mar,inc | Complex/High | IP, MSC, IF |
| **ALL14** | 39 | M | Diagnosis | BM | 46,XY,t(9;22)(q34;q11.2),inc | t(9;22)/High | IP, MSC, IF |
| **ALL15** | 36 | M | Diagnosis | BM | 48,XY,+Y,+add(1)(p1),der(19)t(1;19)(q2;p13) | t(1;19)/Standard | IP, MSC, CFU-F, IF, PDX |
| **ALL16** | 52 | M | Diagnosis | BM | 47,XY,+X,t(14;19)(q32;q13) | Other/Standard | MSC, CFU-F, IF |
| **ALL17** | 46 | F | Diagnosis | BM | 40,X,-X,del(1)(q32q42),-3,-7,-13,-15,-16,-17,+mar | Other/Standard | MSC, CFU-F, IF |
| **ALL18** | 49 | F | Diagnosis | BM | [2]Failed | Other/Standard | MSC, IF |
| **ALL19** | 45 | F | Diagnosis | BM | [3]46,XX | Other/Standard | MSC, CFU-F |
| **ALL20** | 48 | F | Diagnosis | BM | 46,XX,t(9;22)(q34;q11.2) | t(9;22)/High | MSC, CFU-F |
| **ALL21** | 33 | F | Diagnosis | BM | 46,XX | Standard | MSC, CFU-F |
| **ALL22** | 26 | M | Diagnosis | BM | [4]Unavailable | t(4;11)/High | MSC |
| **ALL23** | 30 | M | Diagnosis | BM | 54,XY,+X,+2,+4,+6,t(9;22)(q34;q11.2),+10,+14,+21, +der(22)t(9;22) | t(9;22)/High | MSC, CFU-F, PDX |
| **ALL24** | 52 | M | Diagnosis | PB | 46,XY,t(4;11)(q21;q23) | t(4;11)/High | PDX |
| **ALL25** | 48 | M | Diagnosis | PB | 46,XY,t(4;11)(q21;q23)[26]/46,idem,del(6)(q21q23) | t(4;11)/High | PDX |

**Supplementary Table 3. Patient and disease specific features of primary samples used in the present study.** The percentage of blasts as described in diagnostic reports was >80%. [1]Full karyotype incomplete due to poor morphology; [2]FISH identified TCF3 rearrangement; [3]FISH identified CDKN2A deletion; [4]FISH identified MLL rearrangement.

Post Treat: post treatment; AA: adipocyte analysis; IHC: resistant blast immunohistochemistry; IP: MSC immunophenotyping; MSC: mesenchymal stromal cell outgrowth; CFU-F: colony forming unit fibroblast assay; PDX: patient derived xenograft; IF: immunofluorescence.

| shRNA | Oligo Name | Clone ID | Sequence |
|---|---|---|---|
| **CD36** | TRCN0000057000 | NM_000072.2-962s1c1 | CCGGGCCATAATCGACACATATAAACTCGAGTTTATATGTGTCGATTATGGCTTTTTG |
| **Control** | SHC002V | N/A | CCGGCAACAAGATGAAGAGCACCAACTC-GAGTTGGTGCTCTTCATCTTGTTGTTTTT |

**Supplementary Table 4.** Sequences of transduction particles used for CD36 shRNA knockdown.

| Gene | Forward Primer | Reverse Primer |
|---|---|---|
| CD36 | TCTTTCCTGCAGCCCAATG | AGCCTCTGTTCCAACTGATAGTG |
| ACOX1 | GAACTCACCTTCGAGGCTTG | CCACCAGGCCACCATTTA |
| CPT1A | ACACCATCCAGCACTGAGA | TGAGGCTCCGAGGTATTGTC |
| ATP4A | TTAATCGCAAGGATGCCCGT | CAGTTCCGATGGGTCCATGT |
| LSP1 | CAGACTACAGGCTGATGGCG | AGTGGGCCCCAGCAACTC |
| PFKFB4 | CCCACGGGAATTGACACAGA | GCCCACCTTTGAAAGTTCACC |
| ANKRD65 | CCCTTGGATGGACTCCCAGA | CAACACTAGGCCCCTCTGTC |
| SLC12A7 | TTCAGCATGAAGCCGGACC | TTGTTGAGGACGACGCCATT |
| PDE4C | AGCATTTTTCAGGAGCGGGA | TTGGCGTGGTAGTGACCTTC |
| PSAT1 | AAAAACAATGGAGGTGCCGC | GGCTCCACTGGACAAACGTA |
| ASS1 | CCCGAGCCCGAGTGGT | GGGATTGGAGGCGTGAGTTC |
| GPT2 | GATTCCCGGAAGAGAGTCGG | GGCTTTTTGATACCCTCGGTG |
| SLC7A11 | ATGCAGTGGCAGTGACCTTT | CATGGAGCCAAAGCAGGAGA |
| SLC7A5 | CTGCTCATCATCCGGCCTTC | TGAGCAGCAGCACGCA |
| SLC6A9 | GAGCTGGCAGAGGTGTGAAT | GCACCATTCTGTTCTGGGGA |
| HK2 | GCCTCGGTTTCCCAACTCTG | GGTCAACCTTCTGCACTTGG |
| ENO1 | TCAAAAGGTCTCTTCAGAGCTGC | CCATGGGCTGTGGGTTCTAA |
| PKM | TCAGCGCCGGAGGACC | CAGCTGCTGGGTCTGAATGA |
| RRP1B | GGGGGAAGGAGCTTTTAGCA | TCCTTCGTCTTCGCAGCAAT |
| MPV17L2 | CGCGCTGCTCGTCACTAA | CGCAAACATGCTCGCGGA |
| RRS1 | CGGACCCGTCTGTAAACCAA | CTTGACGGAAGGGCAATCCT |
| sXBP-1 | CTGAGTCCGAATCAGGTGCAG | ATCCATGGGGAGATGTTCTGG |
| usXBP-1 | CAGCACTCAGACTACGTGCA | ATCCATGGGGAGATGTTCTGG |
| Total XBP-1 | TGGCCGGGTCTGCTGAGTCCG | ATCCATGGGGAGATGTTCTGG |
| ATF4 | GTTCTCCAGCGACAAGGCTA | ATCCTGCTTGCTGTTGTTGG |
| CHOP | AGAACCAGGAAACGGAAACAGA | TCTCCTTCATGCGCTGCTTT |
| BiP | TGTTCAACCAATTATCAGCAAACTC | TTCTGCTGTATCCTCTTCACCAGT |
| EDEM | CAAGTGTGGGTACGCCACG | AAAGAAGCTCTCCATCCGGTC |
| b-Actin | GCCGCCAGCTCACCAT | TCGTCGCCCACATAGGAATC |

**Supplementary Table 5.** Forward and reverse sequences of primers used in the present study.

## Supplementary References


1. Dominici M, Le Blanc K, Mueller I, Slaper-Cortenbach I, Marini F, Krause D, et al. Minimal criteria for defining multipotent mesenchymal stromal cells. The International Society for Cellular Therapy position statement. *Cytotherapy.* 2006;8(4):315-7.
2. Ye H, Adane B, Khan N, Sullivan T, Minhajuddin M, Gasparetto M, et al. Leukemic Stem Cells Evade Chemotherapy by Metabolic Adaptation to an Adipose Tissue Niche. *Cell Stem Cell.* 2016;19(1):23-37.
3. Shafat MS, Oellerich T, Mohr S, Robinson SD, Edwards DR, Marlein CR, et al. Leukemic blasts program bone marrow adipocytes to generate a protumoral microenvironment. *Blood.* 2017;129(10):1320-32.
4. Zhang T, Yang J, Vaikari VP, Beckford JS, Wu S, Akhtari M, et al. Apolipoprotein C2 - CD36 Promotes Leukemia Growth and Presents a Targetable Axis in Acute Myeloid Leukemia. *Blood Cancer Discov.* 2020;1(2):198-213.
