# Supplementary material for: Adipocytes disrupt the translational programme of acute lymphoblastic leukaemia to favour tumour survival and persistence
Source: Nat Commun. 2021 Sep 17;12:5507. doi: 10.1038/s41467-021-25540-4 (PMC8448863; doi:10.1038/s41467-021-25540-4)
Supplement: Supplementary file 2 — Reporting summary [file 41467_2021_25540_MOESM2_ESM.pdf]

nature research

Corresponding author(s): Bela Patel
Last updated by author(s): 25/05/2020

Reporting Summary

Nature Research wishes to improve the reproducibility of the work that we publish. This form provides structure for consistency and transparency in reporting. For further information on Nature Research policies, see our Editorial Policies and the Editorial Policy Checklist.

Statistics

For all statistical analyses, confirm that the following items are present in the figure legend, table legend, main text, or Methods section.

n/a	Confirmed	
☐	☒	The exact sample size (n) for each experimental group/condition, given as a discrete number and unit of measurement
☐	☒	A statement on whether measurements were taken from distinct samples or whether the same sample was measured repeatedly
☐	☒	The statistical test(s) used AND whether they are one- or two-sided<br>*Only common tests should be described solely by name; describe more complex techniques in the Methods section.*
☐	☒	A description of all covariates tested
☐	☒	A description of any assumptions or corrections, such as tests of normality and adjustment for multiple comparisons
☐	☒	A full description of the statistical parameters including central tendency (e.g. means) or other basic estimates (e.g. regression coefficient) AND variation (e.g. standard deviation) or associated estimates of uncertainty (e.g. confidence intervals)
☒	☐	For null hypothesis testing, the test statistic (e.g. F, t, r) with confidence intervals, effect sizes, degrees of freedom and P value noted<br>*Give P values as exact values whenever suitable.*
☐	☒	For Bayesian analysis, information on the choice of priors and Markov chain Monte Carlo settings
☐	☒	For hierarchical and complex designs, identification of the appropriate level for tests and full reporting of outcomes
☐	☒	Estimates of effect sizes (e.g. Cohen's d, Pearson's r), indicating how they were calculated

Our web collection on statistics for biologists contains articles on many of the points above.

Software and code

Policy information about availability of computer code

Data collection	not applicable
Data analysis	not applicable

For manuscripts utilizing custom algorithms or software that are central to the research but not yet described in published literature, software must be made available to editors and reviewers. We strongly encourage code deposition in a community repository (e.g. GitHub). See the Nature Research guidelines for submitting code & software for further information.

Data

Policy information about availability of data

All manuscripts must include a data availability statement. This statement should provide the following information, where applicable:
- Accession codes, unique identifiers, or web links for publicly available datasets
- A list of figures that have associated raw data
- A description of any restrictions on data availability

Now provided as a subheading in the main manuscript

Field-specific reporting

Life sciences study design

All studies must disclose on these points even when the disclosure is negative.

Sample size	No formal sample size calculations were performed. The experimental designs were based on known biological variance of the cell lines and statistical feasibility. In the case of in vivo work, previous experience with engraftment success was used to calculate appropriate cohort size whilst minimising animal excess.
Data exclusions	No data exclusions to declare
Replication	Where feasible, reproducibility was confirmed by ensuring appropriate numbers of technical replicates in addition to performing independent experiments. For primary specimens, multiple biologically independent tissues were assessed
Randomization	Randomisations were not applicable for the design of our experiments
Blinding	Although formal blinding was not performed due to the nature of the studies involved, technical execution of experiments were largely separate to data analysis thereby avoiding a differential treatment effect at the outset.

Reporting for specific materials, systems and methods

We require information from authors about some types of materials, experimental systems and methods used in many studies. Here, indicate whether each material, system or method listed is relevant to your study. If you are not sure if a list item applies to your research, read the appropriate section before selecting a response.

Materials & experimental systems

n/a	Involved in the study	
☐	☒	Antibodies
☐	☒	Eukaryotic cell lines
☒	☐	Palaeontology and archaeology
☐	☒	Animals and other organisms
☒	☐	Human research participants
☐	☒	Clinical data
☒	☐	Dual use research of concern

Methods

n/a	Involved in the study	
☒	☐	ChIP-seq
☐	☒	Flow cytometry
☒	☐	MRI-based neuroimaging

Antibodies

Antibodies used

Primary antibodies were purchased from Cell Signaling Technology: P21 (12D1, 2947), P27 (D69C12, 3686), phospho-P53 (Ser46, 2521), Cyclin D1 (E3P5S, 55506), Cyclin E (HE12, 4129), phospho-Rb (Ser807/811, 8516), E2F-1 (3742), Phospho-AMPK (Thr172, 2531), Phospho-mTOR (Ser2448; 2971), Phospho-S6 Ribosomal Protein (Ser235/236; 2211), S6 Ribosomal Protein (5G10; 2217), Phospho-4EBP1 (Thr37/46; 2855), 4EBP1 (53H11; 9644), Phospho-eIF2α (Ser51; 9721), eIF2α (9722) and ATF4 (D4B8; 11815). The anti α-Tubulin antibody was purchased from Abcam (DM1A; ab7291).
CD45-FITC (clone 2D1; 345808, BD Biosciences)
CD10-FITC (clone W8E7; 347503, BD Biosciences)
CD36-APC (clone CB38; 550956, BD Biosciences)
CD10-APC (clone HI10a 332777 BD Biosciences)
CD34-APC (clone ; 555824 BD Biosciences)
CD33-PE (clone WM53; 555450, BD Biosciences)
BODIPY™ 493/503 (4,4-Difluoro-1,3,5,7,8-Pentamethyl-4-Bora-3a,4a-Diaza-s-Indacene) (D3922, Thermo Fisher Scientific)
Ki67-FITC (556026, BD Biosciences)
Fixable Viability Dye eFluor™ 780 (65-0865-14, Thermo Fisher Scientific)
CD19-PE (clone 4G7; 345777, BD Biosciences)
Annexin V-Alexa Fluor 647 (A23204, Thermo Fisher Scientific)
CountBright™ Absolute Counting Beads (C36950, Thermo Fisher Scientific)
DAPI (D3571, Thermo Fisher Scientific).
CD79a (clone SP18, Roche),
CD34 (QBend 10, Roche),
CD22 (clone SP104, Roche),
Tdt (5267811001, Roche)
PAX5 (EPR3730, Abcam,).

Validation

Manufacturer validations were accepted and were further confirmed against the known biology, clinical diagnostic data and performance of the antibody for example against experimental controls and molecular weight ladders.

Eukaryotic cell lines

Policy information about cell lines

Cell line source(s)	Nalm-6 ATCC<br>3T3-L1 (CL-173) from ATCC<br>Nalm-6 from ATCC<br>REH from ATCC<br>RS4;11 from ATCC
Authentication	Low passage cell lines from commercial sources were used for all studies. As such additional authentication was not performed
Mycoplasma contamination	Was serially negative
Commonly misidentified lines<br>(See ICLAC register)	Not applicable

Animals and other organisms

Policy information about studies involving animals; ARRIVE guidelines recommended for reporting animal research

Laboratory animals	immunodeficient NSG strains
Wild animals	Not applicable
Field-collected samples	Not applicable
Ethics oversight	All animal experiments were performed under license PPL 70/8540 approved by the Home Office of the United Kingdom and in accordance with institutional guidelines. Clinical samples and data collection for research use were approved under NRES Committee London; City & East Research Ethics Committee 10/H0704/65.

Note that full information on the approval of the study protocol must also be provided in the manuscript.

Clinical data

Policy information about clinical studies

All manuscripts should comply with the ICMJEguidelines for publication of clinical research and a completedCONSORT checklist must be included with all submissions.

Clinical trial registration	Not applicable
Study protocol	Not applicable
Data collection	Not applicable
Outcomes	Not applicable

Flow Cytometry

Plots

Confirm that:

- [ ] The axis labels state the marker and fluorochrome used (e.g. CD4-FITC).
- [x] The axis scales are clearly visible. Include numbers along axes only for bottom left plot of group (a 'group' is an analysis of identical markers).
- [x] All plots are contour plots with outliers or pseudocolor plots.
- [x] A numerical value for number of cells or percentage (with statistics) is provided.

Methodology

Sample preparation	Single cell suspensions were subjected to Antibody staining performed for 15 min, protected from light. DAPI (1:2,000 in DPBS containing 2% FBS) was added prior to flow cytometric analysis.
Instrument	LSRFortessa™ flow cytometer (BD Biosciences). Data were analysed by FlowJo (version 10.1, Tree Star).
Software	Data were analysed by FlowJo (version 10.1, Tree Star).
Cell population abundance	Not applicable

Gating strategy

Gates were set up to exclude doublets, non-viable cells (DAPI+ or Fixable Viability Dye+) and isotype-stained populations.

☒ Tick this box to confirm that a figure exemplifying the gating strategy is provided in the Supplementary Information.
